# Research progress on phytochemistry and pharmacology of *Staphylea arguta* (Lindl.) Byng & Christenh. and prediction of quality markers: a review

**DOI:** 10.3389/fphar.2025.1684371

**Published:** 2025-10-15

**Authors:** Hongjing Cui, Zhenzhen Wei, Chunyan Feng, Xiuhua Tao, Jianfeng Yi

**Affiliations:** ^1^ Institute for Advanced Study, Jiangxi University of Chinese Medicine, Nanchang, China; ^2^ Integrated Chinese and Western Medicine Institute for Children Health and Drug Innovation, Institute of Chinese Medicine, Jiangxi University of Chinese Medicine, Nanchang, China; ^3^ Research Center for Differentiation and Development of Traditional Chinese Medicine Basic Theory, Jiangxi University of Chinese Medicine, Nanchang, China; ^4^ Jiangxi Academy of Forestry Sciences, Nanchang, China

**Keywords:** Staphylea arguta (Lindl.) Byng & Christenh, traditional Chinese medicine, phytochemistry, pharmacology, quality marker

## Abstract

**Background:**

*Staphylea arguta* (Lindl.) Byng & Christenh. [*Staphyleaceae*] (*Staphylea arguta*), a tree or shrub species belonging to the *Staphyleaceae* family, is widely distributed in subtropical regions of China, particularly in Jiangxi Province, where it is recognized as a geo-authentic botanical drug. It thrives in humid, shaded mountainous areas at elevations of 400–700 m. Traditionally, it has been used for treating breast abscesses, paralysis, sore throat, ulcers, and contusions. Despite its widespread use, systematic reviews on its phytochemistry and pharmacology are limited, and quality control remains challenging due to the lack of standardized markers. This review aims to summarize its metabolites and pharmacological activities, and predict potential quality markers (Q-markers) based on the “five principles” of traditional Chinese medicine (TCM) quality control to address these research gaps.

**Methods:**

The metabolites and pharmacological activities of *Staphylea arguta* were reviewed using data retrieved from scientific databases, including PubMed, Web of Science, and China National Knowledge Infrastructure (CNKI). The “five principles” of TCM Q-markers, which include kinship and chemical specificity, traditional medicinal properties, traditional efficacy, metabolites measurability, and different origins, were applied to identify potential candidates for *Staphylea arguta*.

**Results:**

This review summarizes 451 metabolites from *Staphylea arguta,* including flavonoids, terpenoids, volatile oils and other metabolites, which exhibit anti-inflammatory, analgesic, and immunomodulatory effects. In addition, it is predicted that ligustroflavone (no.23), rhoifolin (no.24), corosolic acid-28-O-β-D-glucopyranosyl ester (no.56), 23-hydroxyoleanolic acid (no.77), squalene (no.253), α-copaene (no.225), and additional identified metabolites can serve as Q-markers for *Staphylea arguta*.

**Conclusion:**

This comprehensive review not only consolidates the existing knowledge on *Staphylea arguta* but also proposes a foundation for its quality standardization. The identified Q-markers will facilitate the development of a robust quality control system, ensuring the safety and efficacy of *Staphylea arguta*-based products for clinical use. This work is significant for promoting the rational utilization and further development of this medicinal resource.

## Graphical Abstract

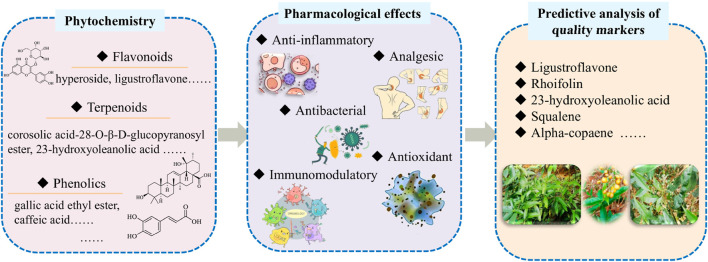

## 1 Introduction

*Staphylea arguta* (Lindl.) Byng & Christenh. [*Staphyleaceae*] (*Staphylea arguta*), locally known as Shanxiangyuan with aliases including Qiandachui (Thousand-Hammer), Qicunding (Seven-Inch Nail), and Eziyao (Moth Medicine), is a tree or shrub species of the genus *Staphylea* in the *Staphyleaceae* family (Institute of Botany, 1972; Commission, 2010). It is naturally distributed across East Asia, including China, Japan, and India, with a predominant presence in the subtropical regions of southern China. In China, it grows mainly in Jiangxi, Hunan, Hubei, Guangxi, and Guizhou provinces, typically in humid, shaded mountainous areas at elevations of 400–700 m. It is recognized as a geo-authentic botanical drug in Jiangxi Province (Wang et al., 2020; Lu et al., 2022). *Staphylea arguta* possesses medicinal value, with its main active metabolites including flavonoids, terpenoids, and volatile oils (Li et al., 2015). It is included in the Pharmacopoeia of the People’s Republic of China (2010 edition) (Commission, 2010) and holds a significant position in both traditional and modern medicine. In traditional Chinese medicine (TCM), the dried leaves are used to clear heat and detoxify (Qingre Jiedu), soothe the throat and reduce swelling (Liyan Xiaozhong), and activate blood circulation to alleviate pain (Huoxue Zhitong). It is clinically applied in the treatment of tonsillitis/pharyngitis, sore throat, skin abscesses, and traumatic injuries. Preparations derived from *Staphylea arguta* leaves include *Staphylea arguta* granules and *Staphylea arguta* baccal tablets. In folk practice, fresh leaves are decocted for oral administration to treat pharyngitis, acute and chronic tonsillitis, peritonsillar abscess, and upper respiratory tract infections, or crushed for topical application on boils and sores, demonstrating rapid efficacy (Li, 2007; Sun, 2008; Zhao et al., 2018; Liu, 2022). As documented in Flora of China, the leaves are applied externally to stop bleeding and heal wounds, or taken orally to treat traumatic injuries (Fang, 1981). Modern studies have confirmed its anti-inflammatory, analgesic, antibacterial, and immunomodulatory properties. Further research on its clinical therapeutic potential and value will enhance its added value and promote its utilization and development (Zhao et al., 2012; Duan and Gao, 2019; Zhang H. W. et al., 2025).

Academician Liu Changxiao proposed the Quality Markers (Q-markers) theory, which was developed by integrating traditional principles of medicinal properties, preparation methods for Chinese patent medicines, and clinical applications of botanical drugs. The Q-markers theory emphasizes the identification of multi-metabolite biomarkers that collectively reflect the quality, efficacy, and consistency of TCM materials and formulations. The theory is centered on the “The effectiveness of TCMs-the material basis for the quality control of the signature metabolites”. Furthermore, it offers research insights aimed at enhancing the efficacy and safety of TCM, as well as improving the quality standards of its products for clinical application (Liu et al., 2016; Zhang T. et al., 2025). To date, limited systematic reviews have been published on *Staphylea arguta*, with most focusing narrowly on specific metabolites or activities. No comprehensive review integrating phytochemical, pharmacological, and quality marker perspectives is available. This review aims to fill this gap by providing a holistic overview and proposing Q-markers to support quality standardization and further research.

## 2 Methods of data acquisition

A comprehensive literature search was conducted using the PubMed, Web of Science, and China National Knowledge Infrastructure (CNKI) databases. The search terms included: “*Staphylea arguta* (Lindl.) Byng & Christenh.“, “*Staphylea arguta”,* “*Turpinia arguta Seem*”, “*Turpiniae Folium*”, “*Shanxiangyuan*”, “*T. arguta*”, “*Qiandachui*”, “*Qicunding*”, “*Eziyao*”, “*Turpinia*”, and “*Turpinia ternata*”. The search was concluded on 1 February 2025. The criteria for admission and disqualification were as follows: (1) Traditional application and pharmacological research of *Staphylea arguta*; (2) The pharmacological mechanisms by which *Staphylea arguta* or its extracts exert anti-inflammatory, antioxidant, immunomodulatory, antibacterial, or analgesic effects; (3) Identification and structural elucidation of the metabolites of *Staphylea arguta*; (4) Qualitative and quantitative evaluation of chemical markers for the quality control of *Staphylea arguta*. Reviews, meta-analyses, case reports, and patents were excluded from the study. Based on the literature collected from the above-mentioned databases, including classic Chinese medicine books, all eligible studies were analyzed and summarized to predict the potential Q-markers of *Staphylea arguta* according to the “Five Principles” of TCM quality control.

## 3 Botanical characteristics

**FIGURE 1:**
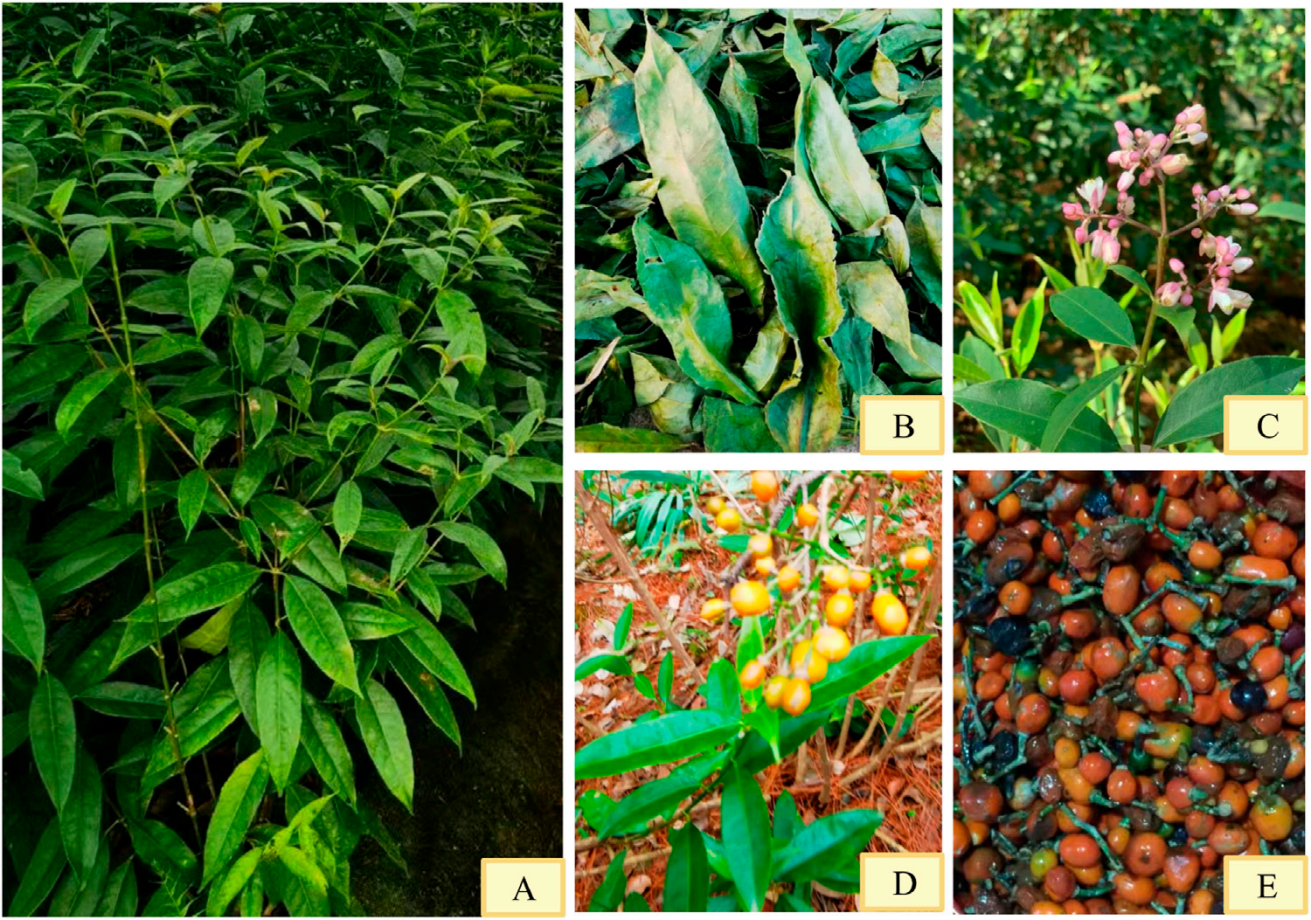
Morphological characteristics of *Staphylea arguta*. **(A)** Shrub. **(B)** Leaves. **(C)** Flowers. **(D)** Fruits. **(E)** Seeds.

*Staphylea arguta* is an evergreen shrub (Figure 1A). Its fruit contains 2 to 26 seeds, with a thousand-seed weight of 20 g. The leaves are opposite and pinnately compound. The leaf axis is about 15 cm long, slender and green (Figure 1B). There are five leaflets, opposite, papery, oblong to oblong-elliptic, measuring (4-)5–6 cm in length and 2–4 cm in width. The leaf tip is caudate-acuminate with a slender tail measuring 5–7 mm, and the base is broadly cuneate. The margins bear sparse rounded teeth or serrations. Both surfaces are glabrous; the upper surface is green, while the lower surface is lighter in color (Figure 1B). There are numerous lateral veins, slightly visible on the upper side and distinct on the lower side. The reticulate veins are barely visible on both surfaces. The lateral leaflets have petiolules 2–3 mm long, while the central leaflet can have a petiolule up to 15 mm long, which is slender and green. The inflorescence is a terminal panicle, with a rachis up to 17 cm long. The flowers are numerous, loosely arranged, and small, about 3 mm in diameter (Figure 1C). The floral buds begin differentiation from late October to early March of the following year, with flowering occurring in mid-to-late March of the following year and the peak flowering period in early to mid-April. The entire differentiation process lasts for 5 months, proceeding centripetally from the outside inward, with the apical flower in the center of the inflorescence differentiating first. There are 5 sepals, glabrous, broadly elliptic, about 1.3 mm long. The petals are 5, elliptic to circular, pubescent or glabrous, about 2 mm long. The filaments are glabrous. The fruit is spherical, 4–7 mm in diameter, with a thin exocarp about 0.2 mm thick (Figure 1D). It has two to three locules, each containing one seed. The general propagation method is seed propagation (Figure 1E) (Li and Li, 2012).

## 4 Phytochemistry

A total of 451 metabolites have been identified in *Staphylea arguta*, including 55 flavonoids (NO.1–55), 76 terpenoids (no.56–131), 46 phenolics (no.132–177), 17 megastigmanes (no.178–194), 9 tannins (no.195–203), 14 alkaloids (no.204–217), 7 phenylpropanoids (no.218–224), 67 volatile oils (no.225–291) and 160 additional identified metabolites (no.292–451). The names of specific metabolites are shown in Supplementary Table 1 of the Appendix.

### 4.1 Flavonoids

**FIGURE 2:**
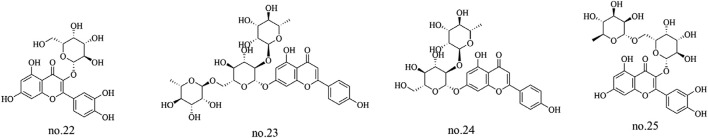
Chemical structure of some flavonoids in *Staphylea arguta*.

Flavonoids are the characteristic and primary active metabolites of *Staphylea arguta*, not only due to their high quantity and diversity but also because of their well-documented biological activities (Li et al., 2015). This class of metabolites includes flavonols, flavanones, isoflavones, and flavonoid glycosides which are classified according to their chemical structure. Argutosides A-E (no.7–11), hyperoside (no.22),ligustroflavone (no.23), rhoifolin (no.24), quercetin-3-O-robinobioside (no.25), and the new flavonoid apigenin-7-(2′-rhamnosyl) rotinoside (no.36) were identified from *Staphylea arguta* (Zhang et al., 2009a; Sun et al., 2012; Ma et al., 2018). The structures of some metabolites are shown in Figure 2.

### 4.2 Terpenoids

**FIGURE 3:**
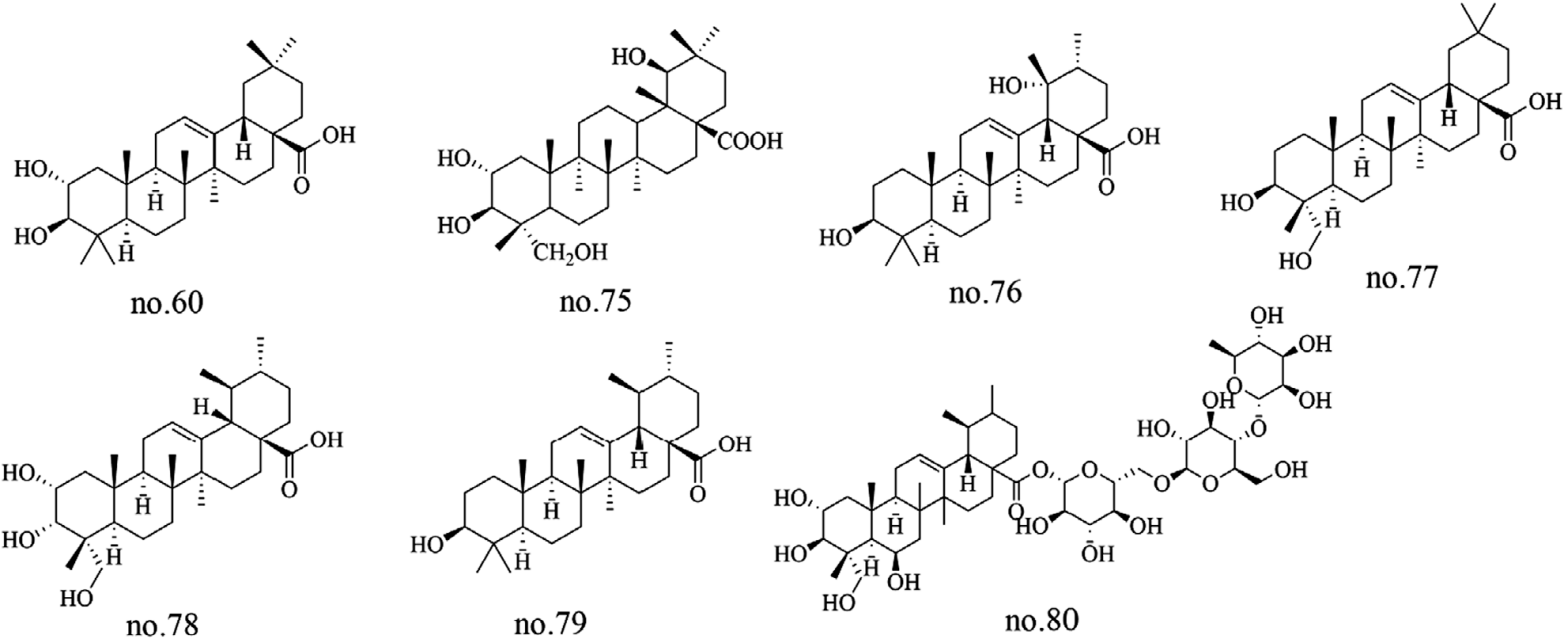
Chemical structures of some terpenoids in *Staphylea arguta*.

Terpenoids represent a major class of natural products in *Staphylea arguta*, with structural diversity primarily encompassing triterpenes and their derivatives. Among the identified terpenoids, several triterpenic metabolites have been isolated and characterized, including corosolic acid-28-O-β-D-glucopyranoside ester (no.56), 2α-peroxyhydroxy ursolic acid (no.60), pomolic acid (no.76), 23-hydroxyoleanolic acid (no.77), 2α,3α,23-trihydroxy ursolic acid (no.78), ursolic acid (no.79), and asiaticoside A (no.80) (Ma et al., 2013; Wu et al., 2012; Kuang et al., 2019). Additionally, 2α,3β,19β-23-tetrahydroxyolean-12-en-28-oic acid (no.75) were identified from *Staphylea arguta* (Huang et al., 2012). The structures of some metabolites are shown in Figure 3.

### 4.3 Phenolics

**FIGURE 4:**
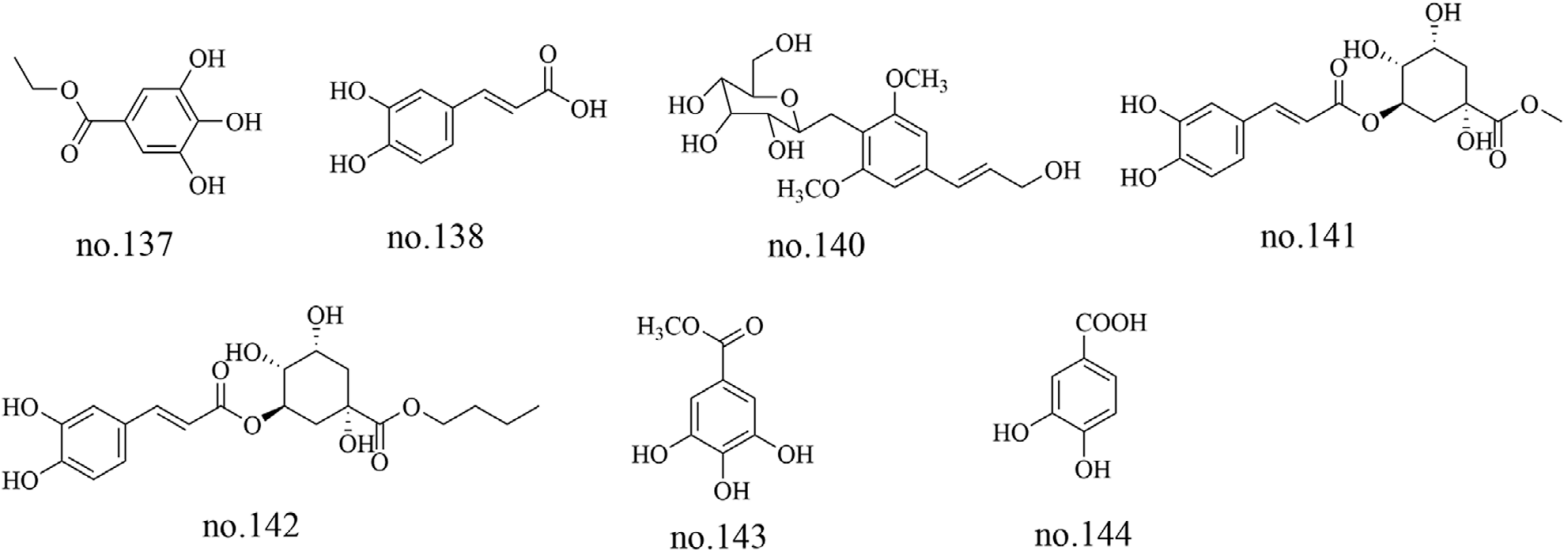
Chemical structure of some phenolics in *Staphylea arguta*.

Based on their structural characteristics, phenolic metabolites can be categorized into phenols, phenylpropanoids, and others. Gallic acid ethyl ester (no.137), caffeic acid (no.138), syringin (no.140), chlorogenic acid methyl ester (no.141), chlorogenic acid butyl ester (no.142), methyl gallate (no.143) and 3,4-dihydroxybenzoic acid (no.144) were identified from *Staphylea arguta* (Wu, 2010; Li et al., 2013; Li et al., 2015). The structures of some metabolites are shown in Figure 4.

### 4.4 Megastigmanes

**FIGURE 5:**
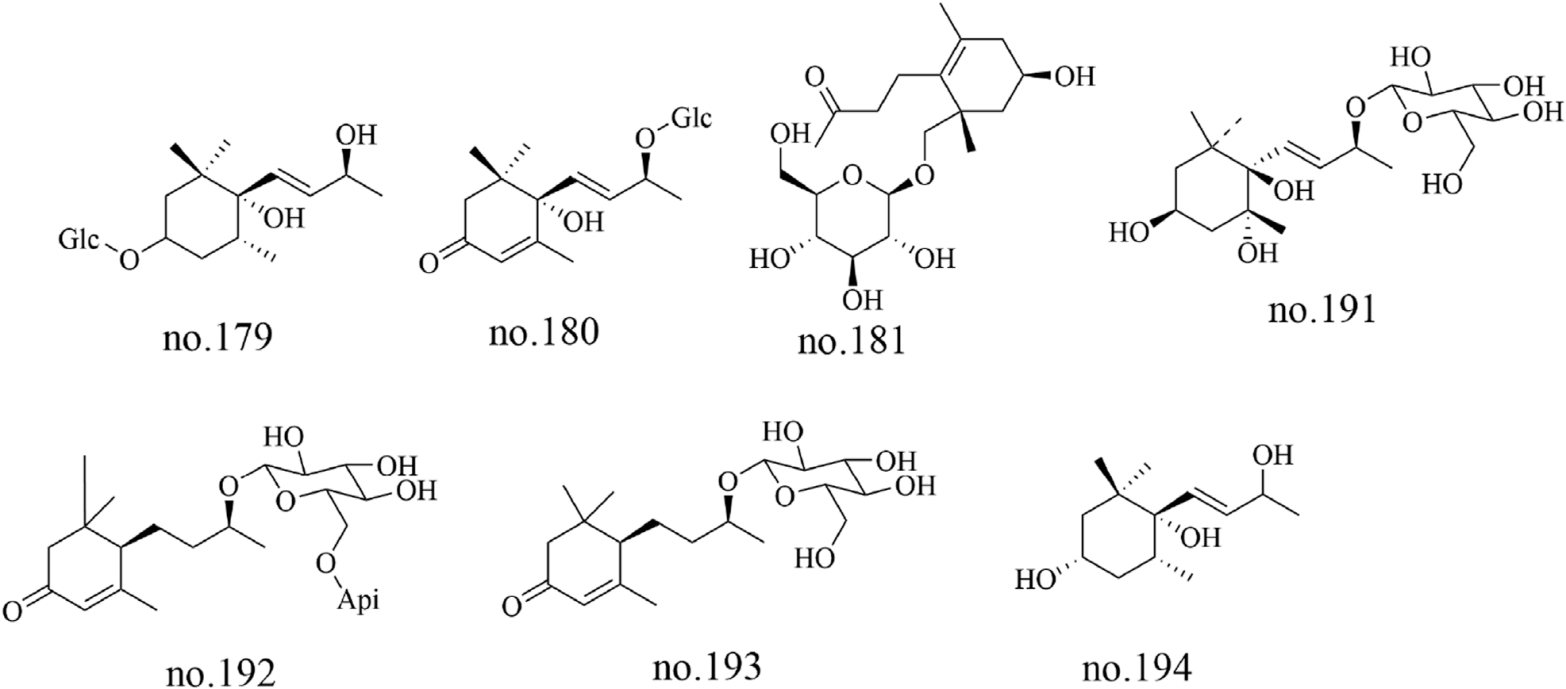
Chemical structure of some megastigmanes in *Staphylea arguta*.

Megastigmanes constitute a class of organic metabolites characterized by distinctive cage-like frameworks. 3S,5R,6R,9S-stetrahydroxy megastigmane (no.179), corchoionoside C (no.180), icariside B4 (no.181), turpinionosides A to E (no.182–186), megastigman-7-ene-3,5,6,9-tetrol-9-O-β-D-glucopyranoside (no.191), byzantionoside B6′-O-β-D-apiofuranoside (no.192), byzantionoside B (no.193) and megastigmene-3,6,9-triol (no.194) were identified from *Staphylea arguta* (Yu et al., 2002; Wu et al., 2014). The structures of some metabolites are shown in Figure 5.

### 4.5 Tannins

**FIGURE 6:**
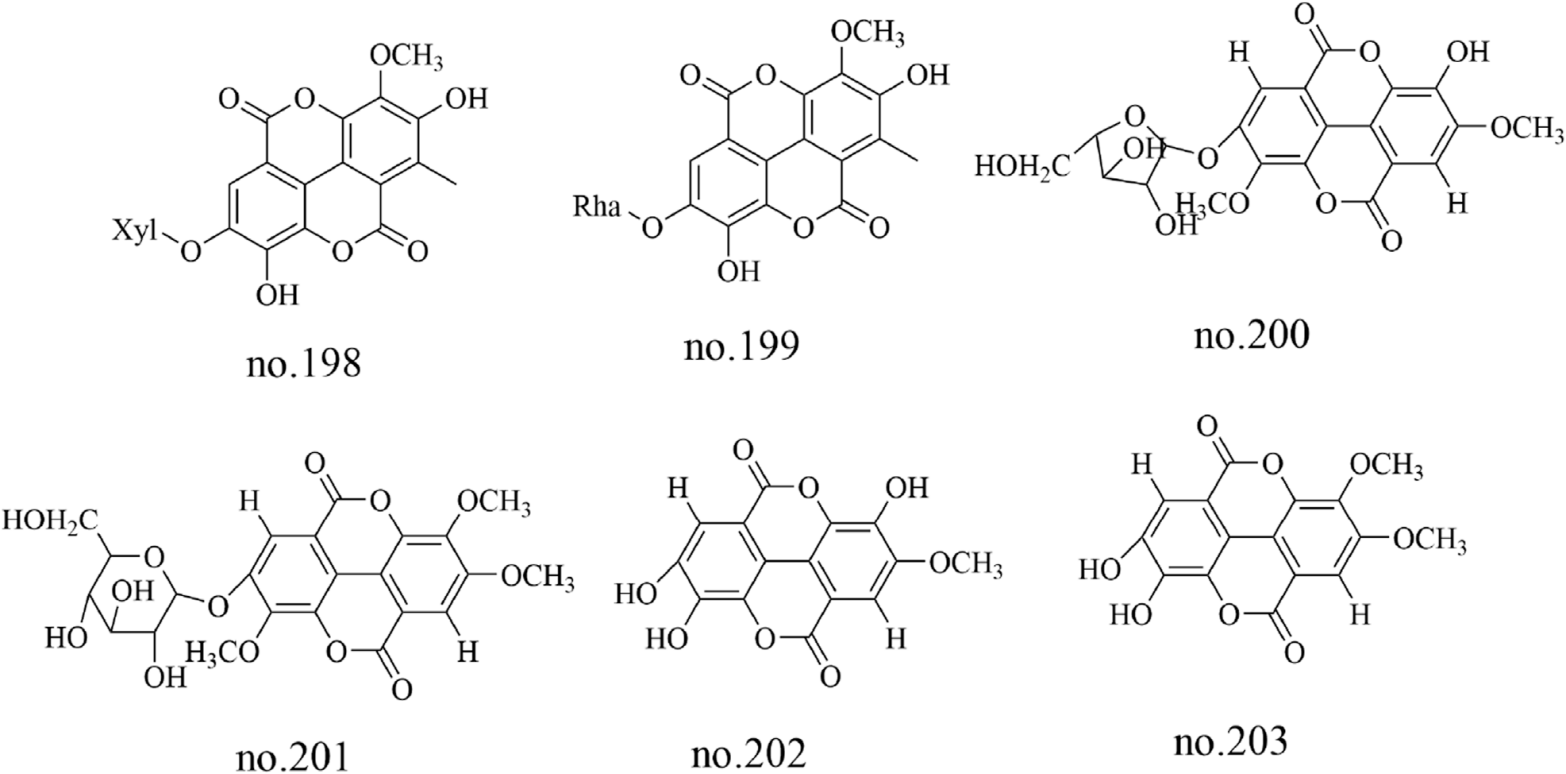
Chemical structure of some tannins in *Staphylea arguta*.

Tannins are a class of polyphenolic metabolites, among which tannin glycosides are the predominant type isolated from *Staphylea arguta*. Specific metabolites identified include 4′-O-methyl ellagic acid-3-O-α-L-rhamnopyranoside (no.195), ellagic acid-3-O-β-D-glucoside (no.196) and ellagic acid-3-O-α-L-rhamnopyranoside (no.197) (Huang et al., 2012; Li et al., 2015). 3′-O-methyl ellagic acid 4-O-β-D-xylopyranoside (no.198), 3′-O-methylellagic acid 4-O-α-L-rhamnopyranoside (no.199),3,4′-di-O-methylellagic acid-4-O-α-L-arabinofuranoside (no.200), 3,3′-di-O-methylellagic acid-4′-O-α-D-glucopyranoside (no.201), ellagic acid (no.202) and 3-O-methylellagic acid (no.203) were identified from *Staphylea arguta* (Matthew et al., 2007; Huang et al., 2012). The structures of some metabolites are shown in Figure 6.

### 4.6 Alkaloids

**FIGURE 7:**
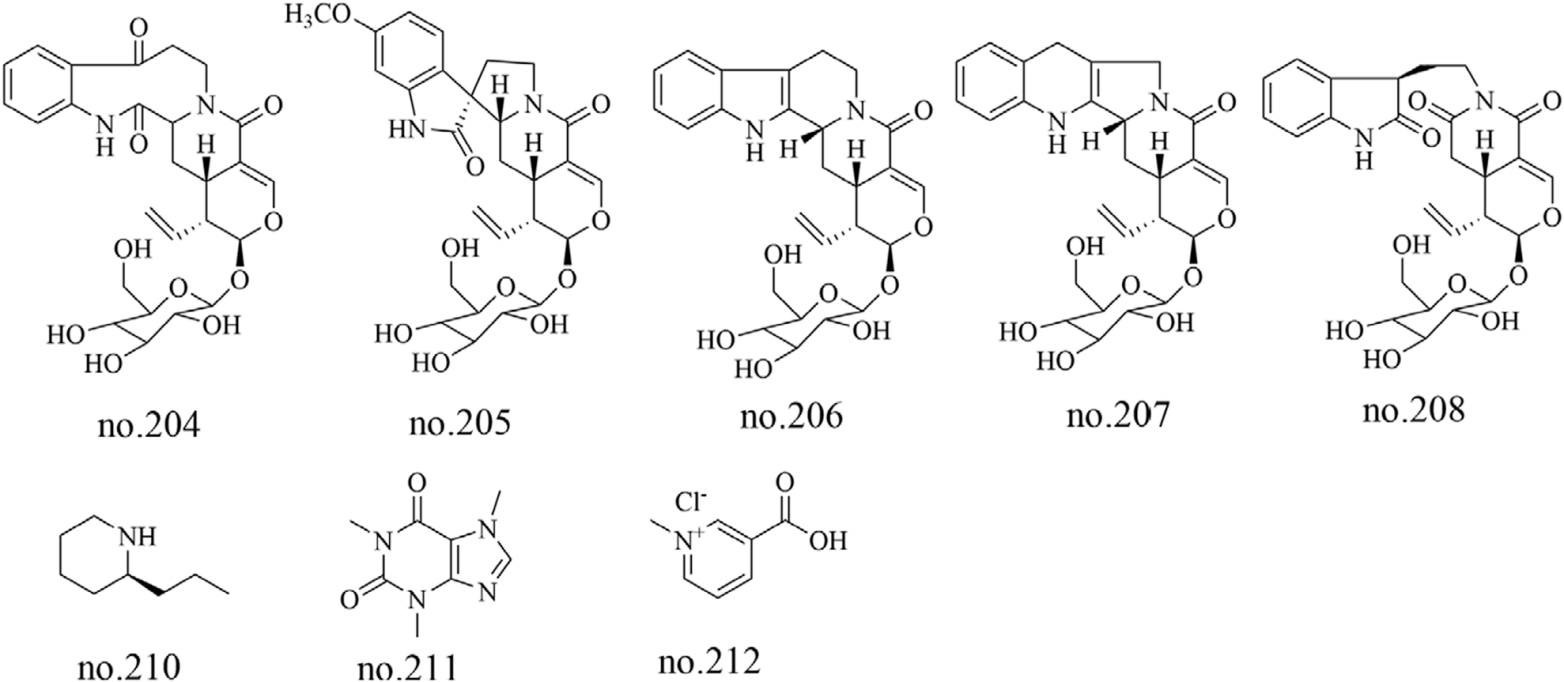
Chemical structure of some alkaloids in *Staphylea arguta*.

Five alkaloids, including 11-methoxyjavaniside (204), vincosamide (205), (3R)-pumiloside (206), turpiniside (207), and paratunamide C (208), were isolated from *Staphylea arguta* (Wu et al., 2011). Liu et al. employed LC-MS to analyze the moderately polar metabolites in its volatile oil, identifying nine alkaloid metabolites including coniine (210), caffeine (211), and trigonelline HCl (212) (Liu et al., 2022). The structures of some metabolites are shown in Figure 7.

### 4.7 Phenylpropanoids

**FIGURE 8:**
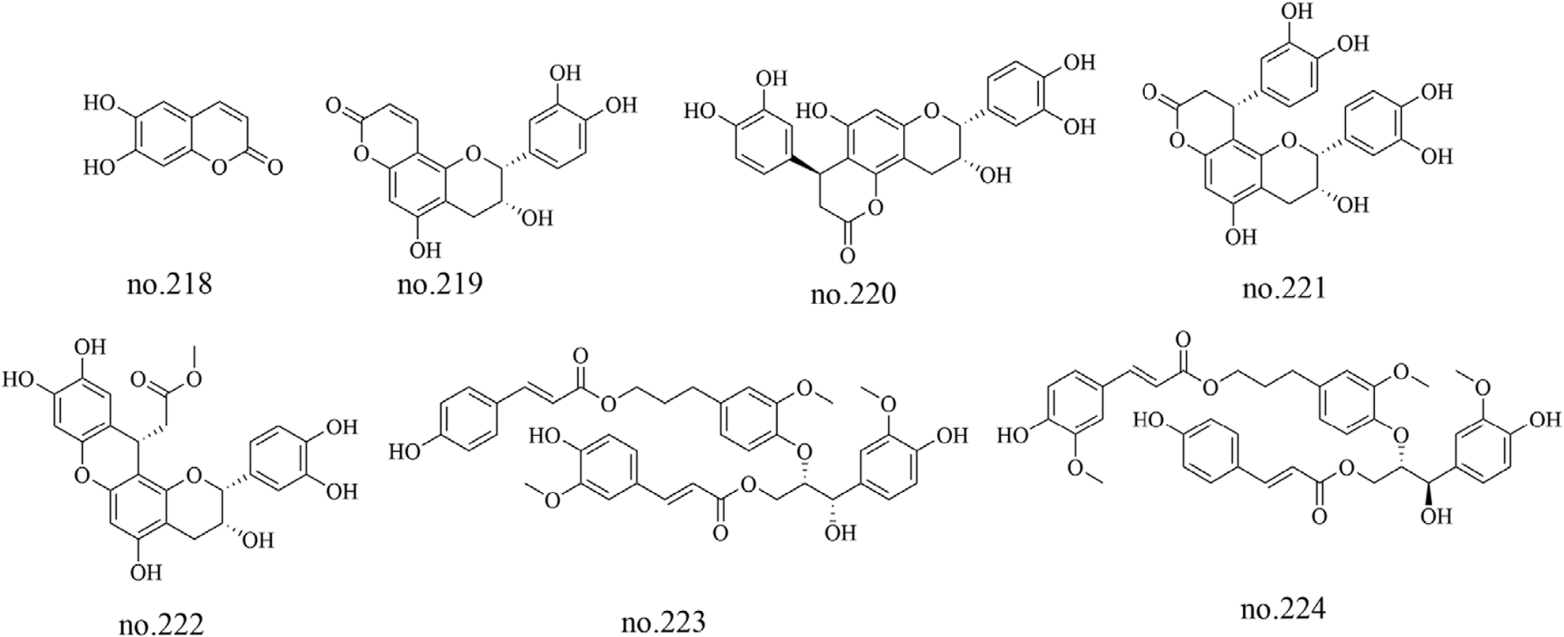
Chemical structure of some phenylpropenoids in *Staphylea arguta*.

Phenylpropanoids, a diverse group of secondary metabolites, have been relatively sparsely documented in *Staphylea arguta*, with only seven such metabolites isolated and identified from the species to date: aesculetin (no.218), epiphyllocoumarin (no.219), cinchonain Ic (no.220), cinchonain Ia (no.221), categuanin B (no.222), turformosin A (no.223) and (−)-(7′S,8′S)-threo-carolignan X (no.224) (Wu, 2010; Huang et al., 2012). The structures of some metabolites are shown in Figure 8.

### 4.8 Volatile oils

Volatile oils, commonly referred to as essential oils, are aromatic plant-derived liquids obtained mainly through distillation. Liu et al. extracted the volatile oil from *Staphylea arguta*, and identified 67 metabolites through GC-MS analysis of low polarity substances, as detailed in Supplementary Table 1 (no.225–291) (Liu et al., 2022).

### 4.9 Additional identified metabolites

**FIGURE 9:**
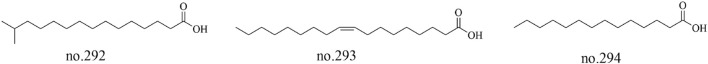
Chemical structure of some fatty acids in *Staphylea arguta*.

**FIGURE 10:**
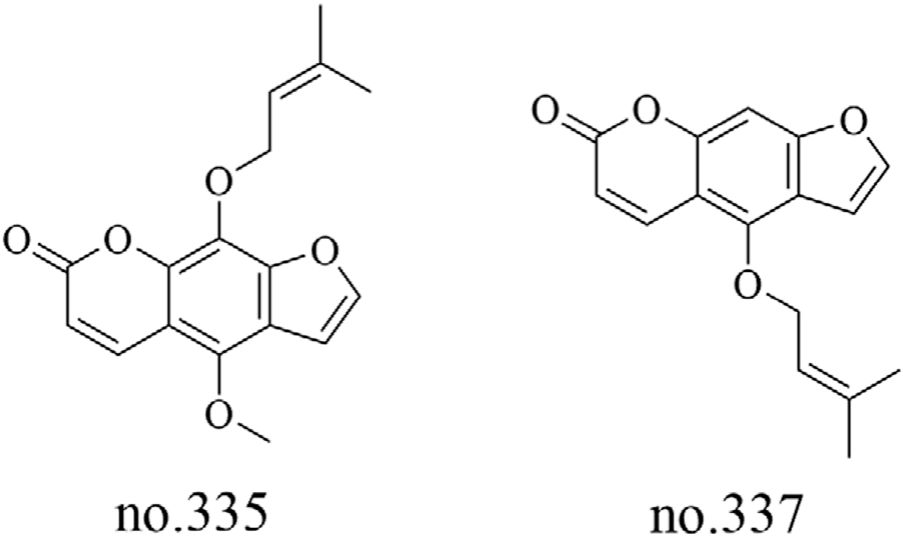
Chemical structure of some coumarins in *Staphylea arguta*.

In addition to the aforementioned metabolites, *Staphylea arguta* also contains fatty acids, coumarins, lactones, vitamins, and other metabolites. Liu et al. utilized LC-MS to analyze the moderately polar metabolites in the volatile oil extracted from *Staphylea arguta*. They identified fatty acid metabolites, including isopalmitic acid (no.292), oleic acid (no.293) and myristic acid (no.294), as well as coumarin metabolites such as phellopterin (no.335) and isoimperatorin (no.337) (Liu et al., 2022). The structures of some metabolites are shown in Figures 9, 10.

## 5 Pharmacological effects

**FIGURE 11:**
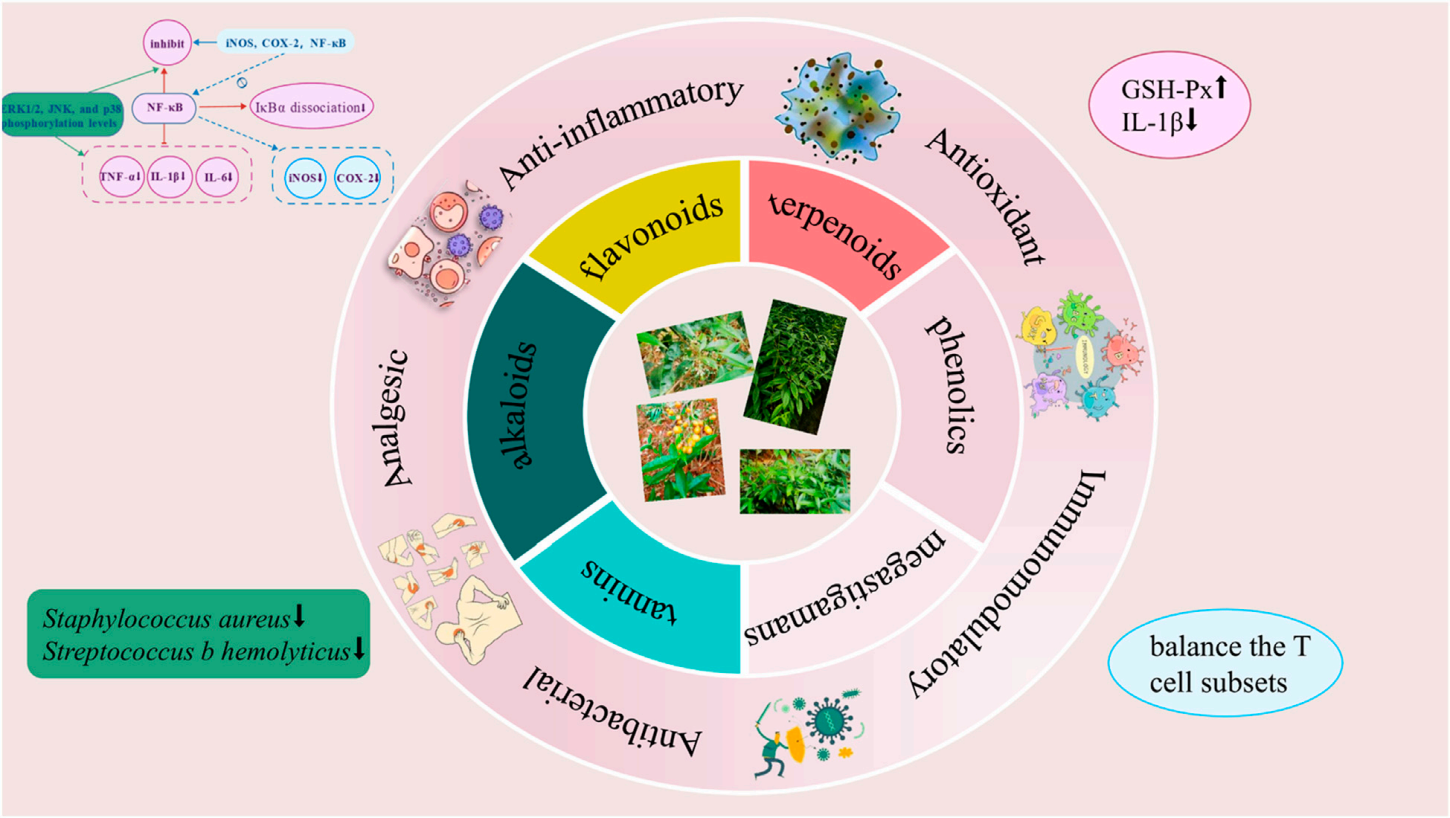
Pharmacological effects of *Staphylea arguta*.

Modern pharmacological studies have demonstrated that *Staphylea arguta* exhibits a range of biological activities, including anti-inflammatory, antioxidant, immunomodulatory, antibacterial, and analgesic effects (Figure 11). Metabolites with pharmacological activities include flavonoids, volatile oils, among others (Table 1).

**TABLE 1:** Pharmacological effects of *Staphylea arguta*.

Activity	Sample	Model/Assay	Dosage	Effect/mechanism	Ref.
Anti-inflammatory	Total flavonoids	Adjuvant arthritis rat model	80, 160, 320 mg/kg	The flavonoids from *Staphylea arguta* can inhibit secondary swelling, polyarthritis, and pathological changes of ankle arthritis in adjuvant-induced arthritis rats	Zhang et al. (2008)
	Total flavonoids	Chronic pharyngitis rat model	0.208 g kg^-1^, 0.416 g kg^-1^, 0.832 g kg^-1^	The total flavonoids from *Staphylea arguta* ameliorate pharyngeal histopathology in the model rats, downregulate the expression of TNF-α, IL-1β, IL-6, and NF-κB p65, and upregulate the expression of IκBα	Chen et al., 2020; Wang (2020)
	Total flavonoids	Mouse monocyte-macrophage leukemic cells RAW 264.7	100, 200, 400 mg L^-1^	The total flavonoids of *Staphylea arguta* inhibit the phosphorylation of ERK1/2, JNK, and p38, and reduce the secretion of TNF-α, IL-1β, and IL-6	Huang et al. (2019)
	Total flavonoids	Mouse monocyte-macrophage leukemic cells RAW 264.7	100, 200, 300 μg mL^-1^	The anti-inflammatory effects of flavonoids are mediated through the inhibition of NF-κB activity and suppression of iNOS and COX-2 expression	Chen et al. (2016)
Antioxidant	Volatile oil	DPPH; ABTS	0.25,0.5,1,2,4,6 mg mL^-1^	The essential oil from the *Staphylea arguta* exhibits significant free radical scavenging activity against both DPPH and ABTS radicals	Liu et al. (2022)
	Total flavonoids, total polysaccharides, polyphenols	GSH-Px	N/A	The extract from *Staphylea arguta* significantly enhanced the activity of GSH-Px	Song et al. (2022)
	Polysaccharides	Hydroxyl radical scavenging, DPPH radical scavenging	N/A	*Staphylea arguta* polysaccharides exhibited their potential as natural antioxidants	Yan et al. (2022)
Immunomodulatory	Total flavonoids	Adjuvant arthritis rat model	10^−4^, 10^−5^, 10^−6^, 10^−7^, 10^−8^ mg L^-1^	The total flavonoids from *Staphylea arguta* regulate the abnormal immune function in adjuvant-induced arthritis rats	Zhang et al. (2007)
	Total flavonoids	Adjuvant arthritis rat model	80, 160, 320 mg kg^-1^	The immunomodulatory effect on the body was related to balancing the T cell subsets	Zhang et al. (2008)
Antibacterial	*Staphylea arguta* baccal tablets	*Staphylococcus aureus* intraperitoneal infection mouse model	5, 10 g kg^-1^	*Staphylea arguta* baccal tablets lowered the death rate in mice with *Staphylococcus aureus* infection	Zhan et al. (2005b)
	*Staphylea arguta* baccal tablets	N/A	N/A	*Staphylea arguta* baccal tablets inhibit *Streptococcus B*	Luo et al. (2003)
	The extract of *Staphylea arguta*	*In vitro* experiment	N/A	The extract of *Staphylea arguta* exhibits an inhibitory effect on *Escherichia coli*, *Salmonella*, and *Staphylococcus aureus*	Xiong et al. (2025)
Analgesic	*Staphylea arguta* baccal tablets	Mouse acetic acid-induced writhing model	N/A	The analgesic rate of *Staphylea arguta* baccal tablets for pain induced by acetic acid injection into the abdominal cavity was 60.3%	Zhan et al. (2005a)

### 5.1 Anti-inflammatory effects

The anti-inflammatory effects of *Staphylea arguta* may be achieved through the inhibition of inflammatory cell activation and the reduction of inflammatory mediator release (Xu, 2011). Zhang et al. investigated the anti-inflammatory effects of total flavonoids extracted from *Staphylea arguta* through animal experiments, and the results demonstrated that these flavonoids could significantly suppress inflammatory responses (Zhang et al., 2007; Zhang et al., 2008). Chen et al. and Wang et al. investigated the mechanism of action of total flavonoids from *Staphylea arguta* in the treatment of chronic pharyngitis. The results demonstrated that the anti-inflammatory mechanism was associated with the inhibition of nuclear factor kappa-B (NF-κB) expression and reduction in nuclear factor kappa B inhibitor alpha (IκBα) dissociation, and downregulation of inflammatory factors such as Tumor necrosis factor α (TNF-α), Interleukin-1β (IL-1β), and Interleukin-6 (IL-6) (Chen et al., 2020; Wang, 2020). Huang et al. investigated the effect of total flavonoids from *Staphylea arguta* on Mitogen-activated protein kinase (MAPK) signaling pathway. The results demonstrated that the anti-inflammatory mechanism was associated with the inhibition of extracellular signal regulated protein kinases 1/2 (ERK1/2), c-Jun N-terminal kinase (JNK), and p38 mitogen-activated protein kinase (p38) phosphorylation levels as well as the reduction in TNF-α, IL-1β, and IL-6 secretion (Huang et al., 2019). Chen et al. reported that total flavonoids from *Staphylea arguta* inhibited lipopolysaccharide-induced inducible nitric oxide synthase (iNOS), Cyclooxygenase-2 (COX-2) and NF-кB expression in RAW264.7 cells. The results demonstrated that the mechanism of its anti-inflammatory effect was associated with the downregulation of NF-кB activity and the suppression of iNOS and COX-2 expression (Chen et al., 2016).

### 5.2 Antioxidant effects

*Staphylea arguta* possess antioxidant properties. Liu et al. assessed the antioxidant effect of volatile oil of *Staphylea arguta* utilizing both 1,1-diphenyl-2-picrylhydra-zyl radical (DPPH) and 2,2′-Azino-bis (3-ethylbenzthiazo-line-6-sulfonic acid), diammonium salt (ABTS) methods. The results indicate that the volatile oil of *Staphylea arguta* exhibits significant antioxidant properties. However, its scavenging ability towards ABTS radicals is comparatively weaker than that towards DPPH radicals (Liu et al., 2022). Song et al. investigated the effects of incorporating *Staphylea arguta* extract to diets on the antioxidant function of Wenchang chickens. The results demonstrated that the extract from *Staphylea arguta* significantly enhanced the activity of glutathione peroxidase (GSH-Px) and markedly reduced the serum IL-1β levels in Wenchang chickens (Song et al., 2022). *Staphylea arguta* polysaccharides exhibited significant hydroxyl radical scavenging, DPPH radical scavenging activities, suggesting their potential as natural antioxidants (Yan et al., 2022).

### 5.3 Immunomodulatory effects

*Staphylea arguta* possess immunomodulatory effects. Zhang et al. investigated the impact of total flavonoids from *Staphylea arguta* on immune function in rats with adjuvant arthritis. The results demonstrated that total flavonoids extracted from *Staphylea arguta* could enhance the compromised immune function of this model rats (Zhang et al., 2007). Zhang et al. studied the mechanism of action of total flavonoids from *Staphylea arguta* in the treatment of adjuvant arthritis. The results showed that the immunomodulatory effect on the body was related to balancing the T cell subsets (Zhang et al., 2008).

### 5.4 Antibacterial effects

Zhan et al. conducted antibacterial experiments on *Staphylea arguta* baccal tablets and found that they significantly reduced the mortality rate of mice infected with *Staphylococcus aureus* (Zhan et al., 2005b; Zhang H. W. et al., 2025). Luo et al. investigated the antibacterial effects of *Staphylea arguta* and demonstrated their ability to significantly inhibit *Streptococcus B* (Luo et al., 2003). Xiong et al. discovered in an *in vitro* study that the extract of *Staphylea arguta* exhibits an inhibitory effect on *Escherichia coli*, *Salmonella*, and *S. aureus* (Xiong et al., 2025).

### 5.5 Analgesic effects

*Staphylea arguta* possesses anti-inflammatory and analgesic properties. Zhan et al. investigated the effects of *Staphylea arguta* baccal tablets on pain responses induced by acetic acid injection into the abdominal cavity, hot plate tests, ear swelling and toe swelling caused by xylene, and increased abdominal capillary permeability due to H^+^. The results indicated that the analgesic rate of *Staphylea arguta* baccal tablets for pain induced by acetic acid injection into the abdominal cavity was 60.3% (Zhan et al., 2005a).

## 6 Research status of Q-markers

Current research in the quality control of TCM comprehensively considers multiple factors, including the growing environment, geographical origin, metabolites, and pharmacological effects of botanical drugs. The complex nature of TCM constitutes the core challenge in quality control studies, as it contains numerous metabolites, each potentially critical to its therapeutic efficacy. Therefore, rapid profiling of crude botanical drug extracts using liquid chromatography-high-resolution mass spectrometry enables metabolite identification; gas chromatography-mass spectrometry facilitates the analysis of volatile metabolites such as essential oils and fatty acids; isolation and purification of individual metabolites are achieved through techniques like column chromatography and preparative liquid chromatography, followed by precise structural elucidation using nuclear magnetic resonance and mass spectrometry. Academician Liu Changxiao proposed the innovative concept of “Q-marker”, which is based on five principles - kinship and chemical specificity, traditional medicinal properties, traditional efficacy, metabolites measurability, and different origins - providing a novel framework for quality control TCM (Liu et al., 2016). Building on this, Gao et al. integrated the clinical applications and pharmacological effects of Shenling Baizhu San, utilizing the five core principles of Q-markers to predict potential Q-markers for this botanical drug in the treatment of respiratory diseases (Gao et al., 2025). Similarly, Luo et al. combined phytochemical and pharmacological studies of the Miao medicine Tiekuaizi, applying the same principles to identify its potential Q-markers (Luo et al., 2024). This comprehensive approach has significantly enhanced the scientific rigor and applicability of quality assessment in TCM.

## 7 Q-markers prediction analysis

**FIGURE 12:**
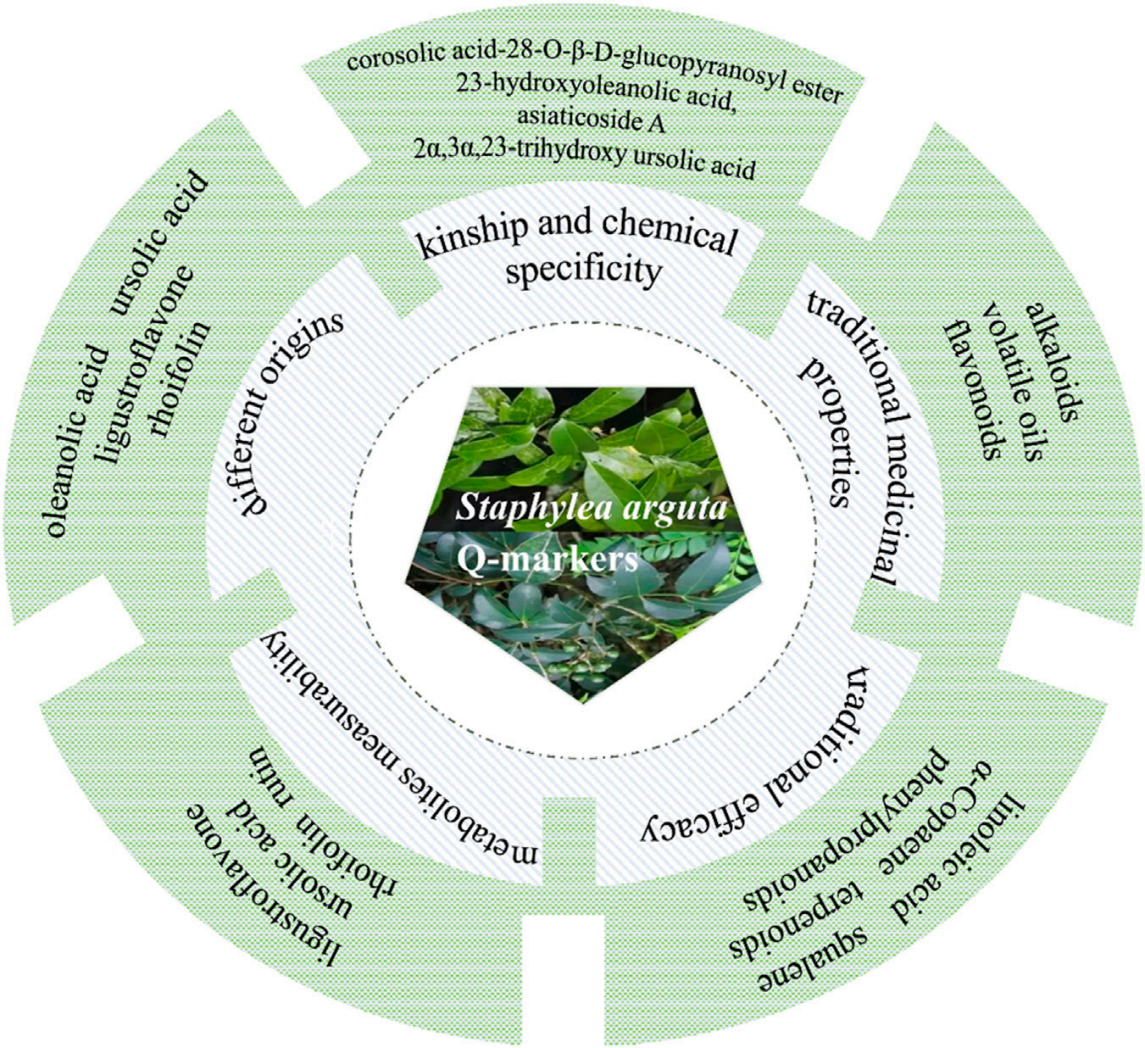
Q-markers prediction analysis of *Staphylea arguta*.

TCM contains a complex and diverse array of metabolites, with clinical efficacy often resulting from synergistic interactions among them. Consequently, quality control based on single-metabolite indicators is insufficient for botanical drugs. The 2020 edition of the Chinese Pharmacopoeia currently designates only ligustroflavone (no.23) and rhoifolin (no.24) as quality control markers for *Staphylea arguta*, which inadequately reflects its comprehensive quality profile (Commission, 2020). To address the limitations of existing quality standards, this study aims to identify potential Q-markers for *Staphylea arguta* based on the Q-marker concept proposed by Academician Liu Changxiao (Liu et al., 2016; Liu et al., 2024), which encompasses five core principles: kinship and chemical specificity, traditional medicinal properties, traditional efficacy, metabolites measurability, and different origins. The objective is to establish a scientifically robust quality evaluation system for this botanical drug (Figure 12).

### 7.1 Q-markers prediction based on kinship and chemical specificity

*Staphylea arguta* belongs to the genus *Staphylea* within the *Staphyleaceae* family. The family *Staphyleaceae* includes the genera *Tapiscia Oliv.*, *Staphylea Linn.*, *Euscaphis Sieb. et Zucc*., and *Turpinia Vent.*, among others, with approximately 60 species (Duan and Gao, 2019). In China, the *Staphyleaceae* family comprises four genera and 22 species. These species are found across the country, with a higher concentration in southwestern regions. The genus *Staphylea* comprises approximately 30–40 species, predominantly distributed across Japan, North America, India, Sri Lanka, and other regions, with 13 species identified in China. The plants within the genus *Staphylea* are abundant in a diverse array of metabolites, primarily comprising flavonoids, terpenoids, phenylpropanoids, volatile oils, and other metabolites. *Staphylea arguta* also contains flavonoids, terpenoids, and other metabolites, which exhibit pharmacological activities including anti-inflammatory, antibacterial, analgesic, and immunomodulatory effects (Xiao et al., 2019). It has been reported that several metabolites, including corosolic acid-28-O-β-D-glucopyranosyl ester (no.56), 23-hydroxyoleanolic acid (no.77), asiaticoside A (no.80), 2α,3α,23-trihydroxy ursolic acid (no.78), caffeic acid (no.138), 3,4-dihydroxybenzoic acid (no.144), 4-hydroxybenzoic acid (no.165), and chlorogenic acid (no.145) have been isolated from *Staphylea arguta* for the first time (Li et al., 2015; Kuang et al., 2019). Among these metabolites, corosolic acid-28-O-β-D-glucopyranosyl ester (no.56), 23-hydroxyoleanolic acid (no.77), asiaticoside A (no.80), and 2α,3α,23-trihydroxy ursolic acid (no.78) are reported for the first time within the *Staphyleaceae* family. These metabolites can be considered distinctive markers that differentiate *Staphylea arguta* from other genera within the same family. In summary, based on the above Kinship and Chemical Specificity, the following metabolites of *Staphylea arguta* can be regarded as Q-markers: corosolic acid-28-O-β-D-glucopyranosyl ester (no.56), 23-hydroxyoleanolic acid (no.77), asiaticoside A (no.80), and 2α,3α,23-trihydroxy ursolic acid (no.78).

### 7.2 Q-markers prediction based on the traditional medicinal properties

The four natures (cold, hot, warm and cool) and five flavors (spicy, sweet, sour, bitter, and salty) constitute the fundamental attributes of TCM. These findings are the outcomes of extensive clinical practice and in-depth investigation, systematically summarizing the properties and therapeutic effects of botanical drugs. Hence, the characteristics of TCM are pivotal in the identification and screening of Q-markers. According to the Chinese Pharmacopoeia (2020 Edition), *Staphylea arguta* is considered to have a cold nature and a bitter taste, and it is classified as belonging to the lung and liver meridians (Commission, 2020). According to TCM, *Staphylea arguta* is used to clear heat, detoxify, relieve sore throat, and reduce swelling. TCM theory suggests that botanical drugs with a bitter taste, such as *Staphylea arguta*, possess properties that can drain, dry, and strengthen-effects consistent with heat-clearing and detoxifying functions. Bitter-tasting heat-clearing botanical drugs typically contain metabolites such as alkaloids, volatile oils, and flavonoids (Wu and Zhang, 2011; Lv et al., 2022). The flavonoid metabolite rhoifolin (no.24), as a representative metabolite of *Staphylea arguta* with bitter-cold property and lung meridian tropism, exerts anti-inflammatory effects through dual mechanisms. Fang et al. established a lipopolysaccharide-induced acute inflammation model in mice to investigate the effects of rhoifolin (no.24) on pathological damage in lung and liver tissues (Fang et al., 2020). Results demonstrated that rhoifolin (no.24) promoted the recovery of liver and lung injuries in acute inflammatory mice and significantly inhibited the secretion of TNF-α, IL-1β, and IL-6 in serum of rat and mouse models. Cellular experiments revealed that 100 μmol/L rhoifolin (no.24) markedly enhanced cell viability, suppressed the production of TNF-α, IL-6, and IL-1β, downregulated mRNA expression of iNOS and C-C motif chemokine ligand 2 (CCL2), and inhibited phosphorylation of IκBα and IκB kinase beta (IκKβ). This mechanism is highly consistent with the traditional “heat-clearing and detoxifying” efficacy. In summary, based on the traditional medicinal properties of *Staphylea arguta*, its alkaloids, volatile oils, and flavonoids are potential candidates for its Q-markers.

### 7.3 Q-markers prediction based on the traditional efficacy

**FIGURE 13:**
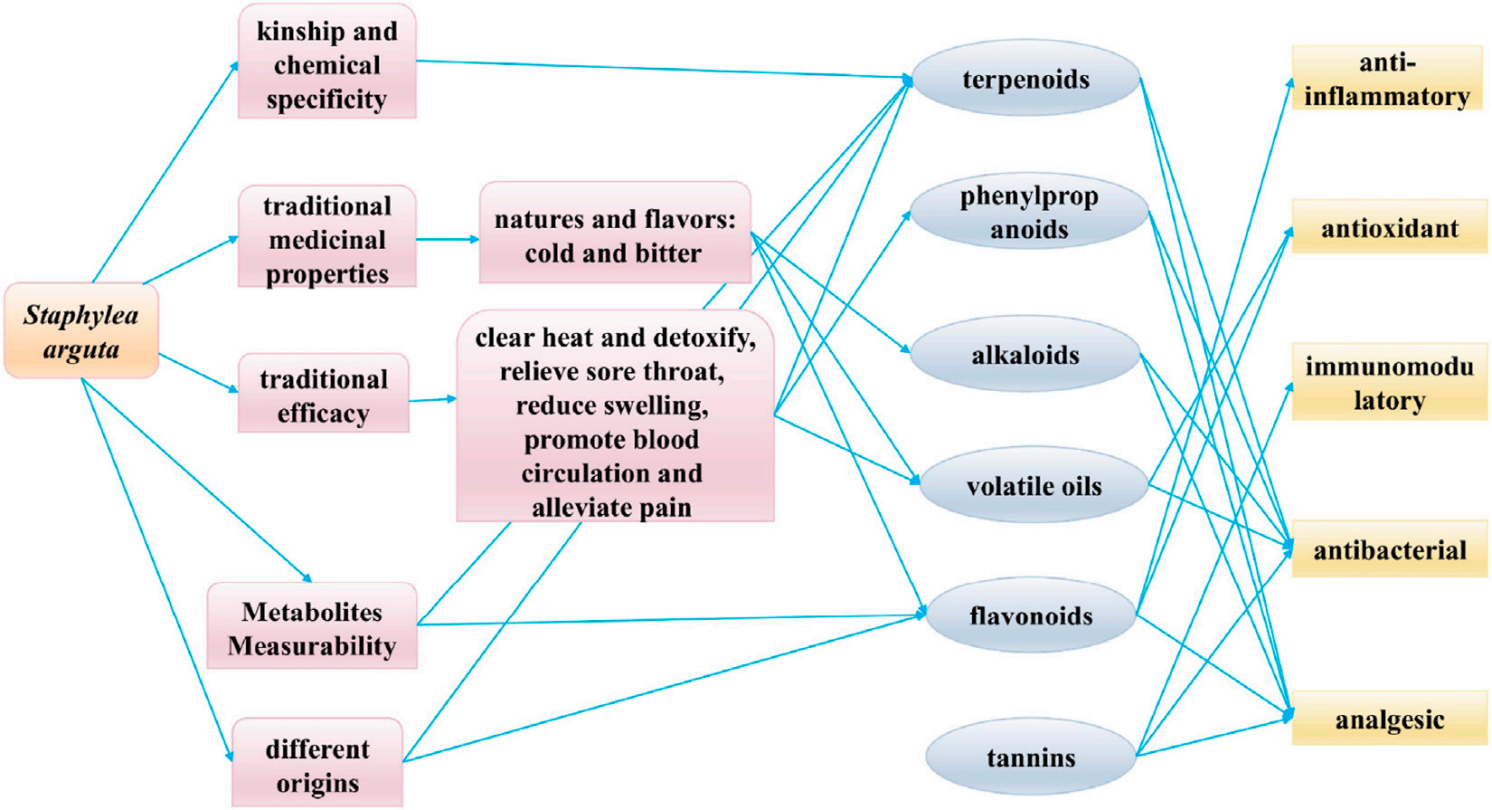
Connections between traditional properties, modern pharmacology, and Q-markers in *Staphylea arguta*.

The established efficacy of traditional medicine serves as the fundamental premise for clinical pharmacotherapy and the cornerstone for ensuring safe drug utilization. “Medicinal properties” and “pharmacodynamic effects” collectively constitute the fundamental elements of the therapeutic efficacy of TCM. The Q-marker theory of TCM proposes a research model based on the ternary relationship of “property-efficacy- metabolite” and the holistic expression of efficacy (Figure 13) (Zhang T. et al., 2025). *Staphylea arguta* possesses the effects of clearing heat and detoxifying, alleviating sore throat and reducing swelling, as well as promoting blood circulation to relieve pain. It is extensively utilized in the treatment of sore throat, ulcerated sore throat, carbuncles and swellings due to toxic heat, as well as traumatic injuries (Zhao et al., 2012; Duan and Gao, 2019). Modern research indicates that linoleic acid (no.240) and squalene (no.253) in *Staphylea arguta* have protective effects on the cardiovascular system (Hildreth et al., 2020; Marangoni et al., 2020; Saini and Rai, 2020). Alpha-Copaene (no.225) demonstrates significant antibacterial and neuroprotective properties (Khoshnazar et al., 2020; Šimunović et al., 2020). Squalene (no.253) exhibits hepatoprotective, renocortical protective, and antitumor properties (Liu et al., 2022). Terpenoids exhibit neuroprotective properties (Suárez Montenegro et al., 2021; Yang et al., 2021). While phenylpropanoids show promising therapeutic potential for neurodegenerative diseases, including Alzheimer’s disease, and associated neuroinflammation (Kolaj et al., 2018; Zhang et al., 2019). In summary, linoleic acid (no.240), squalene (no.253), α-Copaene (no.225), terpenoids, and phenylpropanoids constitute the material foundation upon which *Staphylea arguta* exerts its clinical efficacy. These metabolites can be considered as potential Q-markers for *Staphylea arguta*.

### 7.4 Q-markers prediction based on the metabolites measurability

*Staphylea arguta* is rich in a diverse array of metabolites, and the measurability of these parameters serves as a critical foundation for identifying the Q-markers of TCM. Identifying the active metabolites provides a more comprehensive understanding of the material foundation underlying its pharmacological effects (Liu et al., 2017; Wang et al., 2019). The Chinese Pharmacopoeia specifies the analytical methods and limit requirements for ligustroflavone (no.23) and rhoifolin (no.24) in *Staphylea arguta* (Commission, 2020). To achieve more comprehensively control the quality of *Staphylea arguta*, numerous researchers have chosen methods such as high performance liquid chromatography (HPLC) and ultraviolet-visible spectrophotometry (UV-Vis) for quantitative analysis of *Staphylea arguta*. Liang et al. employed HPLC to quantify the concentration of ligustroflavone (no.23) (Liang et al., 2023). Zhu et al. utilized HPLC to quantify the ursolic acid (no.79) content in *Staphylea arguta* (Zhu et al., 2009). Zhang et al. utilized HPLC to concurrently quantify the concentrations of ligustroflavone (no.23) and rhoifolin (no.24) in *Staphylea arguta* baccal tablets *baccal tablets* (Zhang et al., 2009b). Luo et al. employed UV-Vis spectroscopy to quantify the rutin (no.55) content in *Staphylea arguta* and *Staphylea arguta* oral liquid. The results demonstrated that the aforementioned methods are not only facile to implement but also exhibit superior reproducibility, thereby serving as a valuable reference for the quality control of this botanical drug (Luo et al., 2002a; Luo et al., 2002b). In summary, based on the distinct characteristics of ligustroflavone (no.23), ursolic acid (no.79), rhoifolin (no.24), and rutin (no.55) in *Staphylea arguta*, appropriate methods for determining these characteristic metabolites can be developed, and they can be considered as potential Q-markers for *Staphylea arguta*.

### 7.5 Prediction of Q-markers from different origins based on bioactive metabolites

The quality of botanical drugs varies among different growing regions due to factors such as temperature, humidity, soil composition, and other environmental conditions (Bao et al., 2025). Therefore, controlling the key factors that influence the quality of *Staphylea arguta* is of great significance for ensuring the quality of this botanical drugs. Li et al. utilized HPLC to analyze and compare the content differences of oleanolic acid (no.131) and ursolic acid (no.79) in crude leaf extracts of *Staphylea arguta* collected from 30 different producing areas in Jiangxi, Hunan, Hubei, Guangxi, and other regions. The results indicated that the leaves from Meijiang Town, Xiushan County, Chongqing; Anyuan, Jiangxi; Yudu, Jiangxi; and Taiping Town, Songtao, Guizhou exhibited the highest levels of these two terpenoid metabolites (Li et al., 2022). Xu et al. utilized HPLC to analyze the fingerprint of ligustroflavone (no.23) and rhoifolin (no.24) in crude extracts of *Staphylea arguta* from different sources and found that selecting the common peaks with stable retention time and peak area from each batch to calibrate the fingerprint resulted in a robust chromatographic system, and ligustroflavone (no. 23) could be used as an evaluation index for germplasm resources (Xu and Li, 2017). Liu et al. investigated the concentrations of ligustroflavone (no.23) and rhoifolin (no.24) in crude extracts of *Staphylea arguta* from 10 producing areas and found that the cultivation site with the highest content was the Anyuan base of Jiangxi Shanxiang Pharmaceutical Co., Ltd., which was far higher than the content standards in the 2020 edition of the Chinese Pharmacopoeia (Liu et al., 2012). Yang et al. utilized HPLC to determine the content of ellagic acid (no.202) in crude extracts of *Staphylea arguta* from 10 producing areas and found that the content in the medicinal material produced in Chongqing, Hunan, Hubei, and other regions was higher than that in Jiangxi and Fujian (Yang et al., 2014). Oleanolic acid (no.131), ursolic acid (no.79), ligustroflavone (no.23), rhoifolin (no.24), and ellagic acid (no.202) exhibit content variations correlated with different geographical origins, demonstrating their measurability. Furthermore, oleanolic acid (no.131), ursolic acid (no.79), ligustroflavone (no.23), and rhoifolin (no.24) have been confirmed to associate with traditional medicinal properties and efficacy, supporting their selection as Q-markers.

## 8 Conclusion and perspectives

*Staphylea arguta* represents a medicinally significant plant with considerable therapeutic potential. This review consolidates current knowledge on its phytochemical diversity, pharmacological activities, and Q-marker prediction. A total of 451 metabolites have been identified, encompassing flavonoids, terpenoids, phenolics, and volatile oils, which collectively underpin its broad pharmacological effects. Notably, the plant demonstrates anti-inflammatory, antioxidant, immunomodulatory, antibacterial, and analgesic properties, supporting its traditional uses. Guided by the Q-marker framework, several specific metabolites-including ligustroflavone (no.23), rhoifolin (no.24), corosolic acid-28-O-β-D-glucopyranosyl ester (no.56), 23-hydroxyoleanolic acid (no.77), squalene (no.253), and α-copaene (no.225)-have been proposed as potential Q-markers based on their kinship and chemical specificity, traditional medicinal properties, traditional efficacy, metabolites measurability, and different origins. These Q-markers establish a scientific foundation for the quality control and standardization of *Staphylea arguta*. By identifying metabolites with strong specificity, well-defined efficacy, and measurability as potential Q-markers, this research enables precise quality assessment of TCM. Ultimately, this will contribute to the selection of optimal cultivation regions and ensure batch-to-batch consistency, which proves crucial for developing reliable botanical drugs and validating their traditional applications.

Despite these advances, several research gaps remain. Future studies should prioritize: expanding phytochemical characterization to less-explored metabolites such as alkaloids and phenylpropanoids using advanced UPLC-Q-TOF-MS/MS techniques; elucidating unresolved mechanisms, particularly the molecular targets for immunomodulatory and antibacterial effects; investigating multi-metabolite synergistic effects and conducting robust *in vivo* pharmacokinetic and dose-response studies to validate efficacy and safety; applying network pharmacology, molecular docking, and PBPK modeling to predict multi-target mechanisms and *in vivo* behavior of potential Q-markers. Addressing these aspects will be crucial for advancing the clinical and pharmaceutical application of *Staphylea arguta*, ultimately supporting its standardization and integration into evidence-based TCM.
