# Supplementary material for: Research progress on phytochemistry and pharmacology of Staphylea arguta (Lindl.) Byng & Christenh. and prediction of quality markers: a review
Source: Front Pharmacol. 2025 Oct 15;16:1684371. doi: 10.3389/fphar.2025.1684371 (PMC12568688; doi:10.3389/fphar.2025.1684371)
Supplement: Supplementary file 1 [file Table1.docx]

# Appendix

Table 1 Metabolites of *Staphylea arguta*

| NO. | Mabolites | Source | References |
| --- | --- | --- | --- |
| **flavonoids** | | | |
|  | acacetin 7-O-α-L-rhamnopyranosyl-(1→2)-β-D-glucopyranosyl-(1→6)-β-D-glucopyranoside | A | (Ma et al., 2013) |
|  | acacetin 7-O-α-L-rhamnopyranosyl-(1→2)-α-L-rhamnopyranosy-(1→6)-β-D-glucopyranoside | A | (Ma et al., 2013) |
|  | luteoin 7-O-α-L-rhamnopyranosyl-(1→2)-β-D-glucopyranoside | A | (Ma et al., 2013) |
|  | chrysoeriol 7-O-α-L-rhamnopyranosyl-(1→2)-β-D-glucopyranoside | A | (Ma et al., 2013) |
|  | acacetin 7-O-α-L-rhamnopyranosyl-(1→2)-β-D-glucopyranoside | A | (Ma et al., 2013) |
|  | apigenin-6,8-di-C-β-D-glucopyranoside | A | (Ma et al., 2013) |
|  | argutosides A | A | (Ma et al., 2018) |
|  | argutosides B | A | (Ma et al., 2018) |
|  | argutosides C | A | (Ma et al., 2018) |
|  | argutosides D | A | (Ma et al., 2018) |
|  | argutosides E | A | (Ma et al., 2018) |
|  | apigenin 7-O-triglycoside | A | (Ma et al., 2018) |
|  | apigenin 7-O-α-L-rhamnopyranosyl-(1→2)-[6-O-(3-hydroxy-3-methylglutaryl)]-β-D-glucopyranoside | A | (Ma et al., 2018) |
|  | acacetin 7-O-α-L-rhamnopyranosyl-(1→2)-[6-O-(3-hydroxy-3-methylglutaryl)]-β-D-glucopyranoside | A | (Ma et al., 2018) |
|  | luteolin 7-O-α-L-rhamnopyranosyl-(1→2)-α-L-rhamnopyranosy-(1→6)-β-D-glucopyranoside | A | (Ma et al., 2018) |
|  | luteolin 7-O-α-L-rhamnopyranosyl-(1→2)-[6-O-(3-hydroxy-3-methylglutaryl)]-β-D-glucopyranoside | A | (Ma et al., 2018) |
|  | apigenin 7-O-α-L-rhamnopyranosyl-(1→2)-β-D-glucopyranosyl-(1→6)-β-D-glucopyranoside | A | (Ma et al., 2018) |
|  | apigenin | A;B;C;D | (Li, 2007; Sun, 2008) |
|  | apigenin 7-O-β-D-glucopyranoside | A | (Li, 2007; Sun, 2008) |
|  | luteoin | A | (Li, 2007) |
|  | luteoin 7-O-β-D-glucopyranoside | A | (Li, 2007) |
|  | hyperoside | A | (Zhang et al., 2009a) |
|  | ligustroflavone | A | (Zhang et al., 2009a) |
|  | rhoifolin | A | (Zhang et al., 2009a) |
|  | quercetin-3-O-robinobioside | A | (Zhang et al., 2009a) |
|  | quercetin 3-O-α-L-rhamnopyranosy-(1→6)-β-D-galactopyranoside | A | (Zhang et al., 2009a) |
|  | quercetin | A | (Zhang et al., 2009a) |
|  | narinenin | A | (Li et al., 2013) |
|  | isoliquiritigenin | A | (Li et al., 2013) |

Table 1 (*continued*)

| NO. | Mabolites | Source | References |
| --- | --- | --- | --- |
|  | liquiritigenin | A | (Li et al., 2013) |
|  | aromadendrin | A | (Li et al., 2013) |
|  | quercetin 3-O-α-L-ara-binopyranoside | A | (Li et al., 2015) |
|  | butein | A | (Li et al., 2015) |
|  | acacetin 7-O-β-D-glucopyranoside | A | (Wu, 2010) |
|  | phlorizin | A | (Wu, 2010) |
|  | apigenin-7-(2′-rhamnosyl) rotinoside | A;B;C;D | (Sun, 2008; Sun et al., 2012) |
|  | apigenin-7-O-β-glucoside | A | (Sun et al., 2012) |
|  | naringin | A | (Liu et al., 2022) |
|  | 5-Methoxyflavanone | A | (Liu et al., 2022) |
|  | 5-O-Demethylnobiletin | A | (Liu et al., 2022) |
|  | dichotomitin | A | (Liu et al., 2022) |
|  | noreugenin | A | (Liu et al., 2022) |
|  | trilobatin | A | (Liu et al., 2022) |
|  | naringenin | A | (Liu et al., 2022) |
|  | hydroxygenkwanin | A | (Liu et al., 2022) |
|  | biochanin A | A | (Liu et al., 2022) |
|  | isosakuranetin | A | (Liu et al., 2022) |
|  | 7-Hydroxy-4′-methoxyflavone | A | (Liu et al., 2022) |
|  | bavachalcone | A | (Liu et al., 2022) |
|  | isosakuranin | A | (Liu et al., 2022) |
|  | baicalein | A | (Liu et al., 2022) |
|  | genistein | A | (Liu et al., 2022) |
|  | isoxanthohumol | A | (Liu et al., 2022) |
|  | periplogenin | A | (Liu et al., 2022) |
|  | rutin | A | (Luo et al., 2002a) |
| **terpenoids** | | | |
|  | corosolic acid-28-O-β-D-glucopyranosyl ester | A | (Kuang et al., 2019) |
|  | α-amyrin | A | (Xiao et al., 2019) |
|  | tormentic | A;B;C;D | (Sun, 2008) |
|  | 2α,3β-dihydroxyurs-12-en-28-oic acid | A | (Xiao et al., 2019) |
|  | 2α-peroxyhydroxy ursolic acid | A | (Xiao et al., 2019) |
|  | tormentic acid 28-O-β-D-glucopyranosyl ester | A;B;C;D | (Sun, 2008) |

Table 1 (*continued*)

| NO. | | Mabolites | Source | References |
| --- | --- | --- | --- | --- |
|  | maslinic acid | A;B;C;D | (Sun, 2008) |  |
|  | 2α,3β,19α,23-tetrahydroxyolean-12-en-28-oic acid 28-O-β-D-glucopyranosyl ester | A;B;C;D | (Sun, 2008) |  |
|  | arjunolic acid 28-O-β-D-glucopyranosyl ester | A;B;C;D | (Sun, 2008) |  |
|  | 2α,3β,23,29-tetrahydroxyolean-12-en-28-oic acid 28-O-β-D-glucopyranosyl ester | A;B;C;D | (Sun, 2008) |  |
|  | 3β,6β,23-trihydroxy-12-oleanen-28-oic acid | A | (Sun, 2008) |  |
|  | 3β,6β,23-trihydroxyurs-12-en-28-oic acid | A | (Sun, 2008) |  |
|  | 3β,6β,19α,23-trihydroxyurs-12-en-28-oic acid | A | (Sun, 2008) |  |
|  | 1α,3β,23-trihy droxy-12-oleanen-28-oic acid | A | (Sun, 2008) |  |
|  | arjunglucoside Ⅱ | A | (Sun, 2008) |  |
|  | rosamultin | A | (Sun, 2008) |  |
|  | cinchonagly coside C | A | (Sun, 2008) |  |
|  | mussaendoside S | A | (Sun, 2008) |  |
|  | 3β-O-β-D-glucopyranosylquinovic acid 28-O-β-D-glucopyranosyl ester | A | (Sun, 2008) |  |
|  | 2α,3β,19β-23-tetrahydroxyolean-12-en-28-oic acid | B | (Huang et al., 2012) |  |
|  | pomolic acid | A;B;C;D | (Sun, 2008; Kuang et al., 2019) |  |
|  | 23-hydroxyoleanolic acid | A | (Kuang et al., 2019) |  |
|  | 2α,3α,23 trihydroxy ursolic acid | A | (Kuang et al., 2019) |  |
|  | ursolic acid | A;B;C;D | (Kuang et al., 2019) |  |
|  | asiaticoside A | A | (Kuang et al., 2019) |  |
|  | pechueloic acid | A | (Liu et al., 2022) |  |
|  | meranzin | A | (Liu et al., 2022) |  |
|  | zedoarondiol | A | (Liu et al., 2022) |  |
|  | curcumenol | A | (Liu et al., 2022) |  |
|  | procurcumenol | A | (Liu et al., 2022) |  |
|  | judaicin | A | (Liu et al., 2022) |  |
|  | curcumol | A | (Liu et al., 2022) |  |
|  | camphor | A | (Liu et al., 2022) |  |
|  | (1S,4aR,6aS,6bR,10R,11R,12aR,14bS)-1,10,11-Trihydroxy-2,2,6a,6b,9,9,12a-heptamethyl-1,3,4,5,6,6a,7,8,8a,10,11,12,13,14b-tetradecahydropicene-4a-carboxylic acid | A | (Liu et al., 2022) |  |
|  | ginkgolic acid C17:1 | A | (Liu et al., 2022) |  |
|  | echinenone | A | (Liu et al., 2022) |  |

Table 1 (*continued*)

| NO. | Mabolites | Source | References |
| --- | --- | --- | --- |
|  | cafestol | A | (Liu et al., 2022) |
|  | (1S,2R,4aS,6aS,6bR,10S,12aR)-10-Hydroxy-1,2,6a,6b,9,9,12a-heptamethyl-2,3,4,5,6,6a,7,8,8a,10,11,12,13,14b-tetradecahydro-1H-picene-4a-carboxylic acid | A | (Liu et al., 2022) |
|  | soyasapogenol C | A | (Liu et al., 2022) |
|  | (+)-nootkatone | A | (Liu et al., 2022) |
|  | isosteviol | A | (Liu et al., 2022) |
|  | α-Caryophyllene | A | (Liu et al., 2022) |
|  | (1R,2R,4S,7R,8S,12R)-7-(Furan-3-yl)-1,8,12,17,17-pentamethyl-3,6,16-trioxapentacyclo[9.9.02,4.02,8.012,18]icos-13-ene-5,15,20-trione | A | (Liu et al., 2022) |
|  | dihydroartemisinic acid | A | (Liu et al., 2022) |
|  | asiatic acid | A | (Liu et al., 2022) |
|  | limonin | A | (Liu et al., 2022) |
|  | nardosinone | A | (Liu et al., 2022) |
|  | enoxolone | A | (Liu et al., 2022) |
|  | pristimerin | A | (Liu et al., 2022) |
|  | dihydrocucurbitacin F | A | (Liu et al., 2022) |
|  | 3-[(Carboxycarbonyl)amino]-L-alanine | A | (Liu et al., 2022) |
|  | quillaic acid | A | (Liu et al., 2022) |
|  | ursonic acid | A | (Liu et al., 2022) |
|  | betulin | A | (Liu et al., 2022) |
|  | ginkgolic acid | A | (Liu et al., 2022) |
|  | atractylenolide II | A | (Liu et al., 2022) |
|  | 4,4a,5,6,7,7a,8,9-Octahydro-3,4,8-trihydroxy-6,6,8-trimethylazuleno[5,6-c]furan-1(3H)-one | A | (Liu et al., 2022) |
|  | 14-Deoxy-11,12-didehydroandrographolide | A | (Liu et al., 2022) |
|  | abietic acid | A | (Liu et al., 2022) |
|  | dehydrocostus lactone | A | (Liu et al., 2022) |
|  | andrographolide | A | (Liu et al., 2022) |
|  | citropten | A | (Liu et al., 2022) |
|  | bryodulcosigenin | A | (Liu et al., 2022) |
|  | (5R,9S)-5,9-Dimethyl-14-methylidenetetracyclo[11.2.1.01,10.04,9]hexadecane-5-carboxylic acid | A | (Liu et al., 2022) |
|  | 18β-Glycyrrhetinic acid | A | (Liu et al., 2022) |
|  | zizyberanalic acid | A | (Liu et al., 2022) |

Table 1 (*continued*)

| NO. | | Mabolites | Source | References |
| --- | --- | --- | --- | --- |
|  | echinocystic acid | A | (Liu et al., 2022) |  |
|  | acetyl-11-keto-β-boswellic acid | A | (Liu et al., 2022) |  |
|  | α-Cyperone | A | (Liu et al., 2022) |  |
|  | ingenol-3,4-5,20-diacetonide | A | (Liu et al., 2022) |  |
|  | corosolic acid | A;B;C;D | (Sun, 2008; Liu et al., 2022) |  |
|  | oleanonic acid | A | (Liu et al., 2022) |  |
|  | perillene | A | (Liu et al., 2022) |  |
|  | germacrone | A | (Liu et al., 2022) |  |
|  | α-Santonin | A | (Liu et al., 2022) |  |
|  | oleanolic acid | A | (Li et al., 2022) |  |
| **phenolics** | | | |  |
|  | vanillic acid | A | (Li, 2007) |  |
|  | pyrogallic acid | A | (Li, 2007) |  |
|  | gallic acid | A;B;C;D | (Li, 2007) |  |
|  | eucomic acid | A;B;C;D | (Li, 2007) |  |
|  | E-p-hydroxoy-cinnamic-acid | A | (Li, 2007) |  |
|  | gallic acid ethyl ester | A | (Sun et al., 2012; Li et al., 2013) |  |
|  | caffeic acid | A | (Li et al., 2015) |  |
|  | methoxyhydroquinone-4-β-D-glucopyranoside | B | (Huang et al., 2012) |  |
|  | syringin | A | (Wu, 2010) |  |
|  | chlorogenic acid methyl ester | A | (Wu, 2010) |  |
|  | chlorogenic acid butyl ester | A | (Wu, 2010) |  |
|  | methyl gallate | A | (Wu, 2010) |  |
|  | 3,4-Dihydroxybenzoic acid | A | (Wu, 2010) |  |
|  | chlorogenic acid | A | (Wu, 2010) |  |
|  | methoxyhydroquinone-1-β-D-glucopyranoside | B | (Huang et al., 2012) |  |
|  | D-threo-guaiacylglycerol-O-β-D-glucopyranoside | B | (Huang et al., 2012) |  |
|  | (7S,8R)-syringoylglycerol-7-O-β-D-glucopyranoside | B | (Huang et al., 2012) |  |
|  | 6-O-(p-hydroxybenzoyl)-D-glucose | B | (Huang et al., 2012) |  |
|  | E-glucose-6(4-hydroxy-3-methoxybenzoate) | B | (Huang et al., 2012) |  |
|  | 3-β-O-β-D-glucopyranoylcincholic acid | A | (Wu et al., 2012) |  |
|  | 2,6,10-Trimethyldodecane | A | (Liu et al., 2022) |  |
|  | coumaric acid | A | (Liu et al., 2022) |  |
|  | ferulic acid | A | (Liu et al., 2022) |  |

Table 1 (*continued*)

| NO. | | Mabolites | Source | References |
| --- | --- | --- | --- | --- |
|  | 2-Methoxy-4-(2S,3R)-7-methoxy-3-methyl-5-[(1E)-1-propen-1-yl]-2,3-dihydro-1-benzofuran-2-ylphenol | A | (Liu et al., 2022) |  |
|  | 6-Shogaol | A | (Liu et al., 2022) |  |
|  | veratric acid | A | (Liu et al., 2022) |  |
|  | bergaptol | A | (Liu et al., 2022) |  |
|  | dehydrodiisoeugenol | A | (Liu et al., 2022) |  |
|  | 3,4-Dimethoxybenzaldehyde | A | (Liu et al., 2022) |  |
|  | 2-(Hydroxyimine) pentanedioic acid | A | (Liu et al., 2022) |  |
|  | 3-Hydroxybenzoic acid | A | (Liu et al., 2022) |  |
|  | catechol | A | (Liu et al., 2022) |  |
|  | dihydroferulic acid | A | (Liu et al., 2022) |  |
|  | 4-Hydroxybenzoic acid | A | (Liu et al., 2022) |  |
|  | salicylic acid | A | (Liu et al., 2022) |  |
|  | honokiol | A | (Liu et al., 2022) |  |
|  | 2,4,6-Trihydroxy-5-[1-(4-hydroxy-1,1,4,7-tetramethyl-1a, 2,3,4a, 5,6,7a,7b-octahydrocyclopropa[h]azulen-7-yl)-3-methylbutyl]benzene-1,3-dicarbaldehyde | A | (Liu et al., 2022) |  |
|  | methyl eugenol | A | (Liu et al., 2022) |  |
|  | 4-Nitrophenol | A | (Liu et al., 2022) |  |
|  | 2,3-bis[(4-Hydroxy-3-Methoxyphenyl)methyl]butane-1,4-diol | A | (Liu et al., 2022) |  |
|  | 4-Allylcatechol | A | (Liu et al., 2022) |  |
|  | pyrogallol | A | (Liu et al., 2022) |  |
|  | α,β-Dihydroresveratrol | A | (Liu et al., 2022) |  |
|  | 6-Gingerol | A | (Liu et al., 2022) |  |
|  | magnolol | A | (Liu et al., 2022) |  |
|  | thymol | A | (Liu et al., 2022) |  |
| **megastigmanes** | | | |  |
|  | (2S,9R)-2,9-dihydroxymegastigman-5-en-4-one 2-O-β-D-glucopyranoside | A | (Yu et al., 2002) |  |
|  | 3S,5R,6R,9S-stetrahydroxy megastigmane | A | (Yu et al., 2002) |  |
|  | corchoionoside C | A | (Yu et al., 2002) |  |
|  | icariside B4 | A | (Yu et al., 2002) |  |
|  | turpinionosides A | A | (Yu et al., 2002) |  |
|  | turpinionosides B | A | (Yu et al., 2002) |  |
|  | turpinionosides C | A | (Yu et al., 2002) |  |
|  | turpinionosides D | A | (Yu et al., 2002) |  |

Table 1 (*continued*)

| NO. | Mabolites | Source | References |
| --- | --- | --- | --- |
|  | turpinionosides E | A | (Yu et al., 2002) |
|  | (3S,5R,6S,9S)-3,6,9-trihydroxy megastigman-7-ene 3-O-β-D-glucopyranoside | A | (Yu et al., 2002) |
|  | (3S,5R,6S,9S)-3,6,9-trihydroxy megastigman-7-ene 9-O-β-D-glucopyranoside | A | (Yu et al., 2002) |
|  | (1S,3S,5R,6S,9R)-3,9,12-trihydroxy megastigmane-3-O-β-D-glucopyranoside | A | (Yu et al., 2002) |
|  | (3S,4R,9R)-3,4,9-trihydroxy megastigman-5-ene 3-O-δ-D-glucopyranoside | A | (Yu et al., 2002) |
|  | megastigman-7-ene-3,5,6,9-tetrol-9-O-β-D-glucopyranoside | A | (Wu et al., 2014) |
|  | byzantionoside B6′-O-β-D-apiofuranoside | A | (Wu et al., 2014) |
|  | byzantionoside B | A | (Wu et al., 2014) |
|  | megastigmene-3,6,9-triol | A | (Wu et al., 2014) |
| **tannins** | | | |
|  | 4’-O-methyl ellagic acid-3-O-α-L-rhamnopyranoside | A | (Li et al., 2015) |
|  | ellagic acid-3-O-β-D-glucoside | A | (Li et al., 2015) |
|  | ellagic acid-3-O-α-L-rhamnopyranoside | A | (Li et al., 2015) |
|  | 3’-O-methylellagic acid 4-O-β-D-xylopyranoside | B | (Huang et al., 2012) |
|  | 3’-O-methylellagic acid 4-O-α-L-rhamnopyranoside | B | (Huang et al., 2012) |
|  | 3,4’-di-O-methylellagic acid-4-O-α-L-arabinofuranoside | B | (Matthew et al., 2007) |
|  | 3,3’-di-O-methylellagic acid-4’-O-α-D-glucopyranoside | B | (Matthew et al., 2007) |
|  | ellagic acid | B | (Matthew et al., 2007) |
|  | 3-O-methyl ellagic acid | B | (Matthew et al., 2007) |
| **alkaloids** | | | |
|  | 11-methoxyjavaniside | A | (Wu et al., 2011) |
|  | vincosamide | A | (Wu et al., 2011) |
|  | (3R)-pumiloside | A | (Wu et al., 2011) |
|  | turpiniside | A | (Wu et al., 2011) |
|  | paratunamide C | A | (Wu et al., 2011) |
|  | stachydrine | A | (Liu et al., 2022) |
|  | coniine | A | (Liu et al., 2022) |
|  | caffeine | A | (Liu et al., 2022) |
|  | trigonelline HCl | A | (Liu et al., 2022) |
|  | cantharidin | A | (Liu et al., 2022) |
|  | tazettine | A | (Liu et al., 2022) |

Table 1 (*continued*)

| NO. | Mabolites | Source | References |
| --- | --- | --- | --- |
|  | cinchonine | A | (Liu et al., 2022) |
|  | rhodioloside | A | (Liu et al., 2022) |
|  | 1-[[(2S,3R,11bR)-3-Ethyl-9,10-dimethoxy-2,3,4,6,7,11b-hexahydro-1H-benzo[a]quinolizin-2-yl]methyl]-7-methoxy-3,4-dihydro-2H-isoquinolin-6-one | A | (Liu et al., 2022) |
| **phenylpropanoids** | | | |
|  | aesculetin | A | (Wu, 2010) |
|  | epiphyllocoumarin | A | (Wu, 2010) |
|  | cinchonain Ic | A | (Wu, 2010) |
|  | cinchonain Ia | A | (Wu, 2010) |
|  | categuanin B | A | (Wu, 2010) |
|  | turformosin A | B | (Huang et al., 2012) |
|  | (-)-(7’S,8’S)-threo-carolignan X | B | (Huang et al., 2012) |
| **volatile oils** | | | |
|  | α-Copaene | A | (Liu et al., 2022) |
|  | tetradecane | A | (Liu et al., 2022) |
|  | 2-Hydroxycinnamic acid | A | (Liu et al., 2022) |
|  | germacrene D | A | (Liu et al., 2022) |
|  | 2,4-Di-t-butylphenol | A | (Liu et al., 2022) |
|  | (-)-a-Muurolene | A | (Liu et al., 2022) |
|  | naphthalene,1,2,4a,5,8,8a-hexahydro-4,7-dimethyl-1-(1-methylethyl)-,(1S,4aR,8aS)- | A | (Liu et al., 2022) |
|  | (Z)-Calamanene | A | (Liu et al., 2022) |
|  | (1R)-3α-Vinyl-3-methyl-2β-(1-methylvinyl)-6β-isopropylcyclohexan-1β-ol | A | (Liu et al., 2022) |
|  | heneicosane | A | (Liu et al., 2022) |
|  | hexahydrofarnesyl acetone | A | (Liu et al., 2022) |
|  | palmitic acid | A | (Liu et al., 2022) |
|  | ethyl palmitate | A | (Liu et al., 2022) |
|  | icosane | A | (Liu et al., 2022) |
|  | phytol | A | (Liu et al., 2022) |
|  | linoleic acid | A | (Liu et al., 2022) |
|  | (9Z,12Z)-9,12-Octadecadien-1-yl acetate | A | (Liu et al., 2022) |
|  | ethyl Oleate | A | (Liu et al., 2022) |
|  | octacosyl acetate | A | (Liu et al., 2022) |

Table 1 (*continued*)

| NO. | Mabolites | Source | References |
| --- | --- | --- | --- |
|  | stearoylglycerol | A | (Liu et al., 2022) |
|  | N,N-Dimethyloctanamide | A | (Liu et al., 2022) |
|  | 5-Methyl-5-(4,8,12-trimethyltridecyl)oxolan-2-one | A | (Liu et al., 2022) |
|  | 2,2′-Methylenebis(6-tert-butyl-4-methylphenol) | A | (Liu et al., 2022) |
|  | 1,3-Dihydroxypropan-2-yl hexadec-9-enoate | A | (Liu et al., 2022) |
|  | bis(2-Ethylhexyl) phthalate | A | (Liu et al., 2022) |
|  | tetracontane | A | (Liu et al., 2022) |
|  | octadecamethylcyclononasiloxane | A | (Liu et al., 2022) |
|  | cis-13-Docosenoamide | A | (Liu et al., 2022) |
|  | squalene | A | (Liu et al., 2022) |
|  | hexatriacontane | A | (Liu et al., 2022) |
|  | (6-Chloro-chroman-3-yl)-methylamine | A | (Liu et al., 2022) |
|  | squalane | A | (Liu et al., 2022) |
|  | tetrapentacontane | A | (Liu et al., 2022) |
|  | γ-Sitosterol | A | (Liu et al., 2022) |
|  | 28-Isofucosterol | A | (Liu et al., 2022) |
|  | β-Amyrin acetate | A | (Liu et al., 2022) |
|  | (Z)-Cinnamaldehyde | A | (Liu et al., 2022) |
|  | 1,4-Benzenedicarboxylic acid,dimethyl ester | A | (Liu et al., 2022) |
|  | (-)-α-Muurolene | A | (Liu et al., 2022) |
|  | methyl laurate | A | (Liu et al., 2022) |
|  | (+)-δ-Cadinene | A | (Liu et al., 2022) |
|  | methyl myristate | A | (Liu et al., 2022) |
|  | methyl (9Z)-9-hexadecenoate | A | (Liu et al., 2022) |
|  | methyl hexadec-9-ynoate | A | (Liu et al., 2022) |
|  | methyl palmitate | A | (Liu et al., 2022) |
|  | cis-10-Heptadecenoic acid methyl ester | A | (Liu et al., 2022) |
|  | 15-Methyl palmitic acid methyl ester | A | (Liu et al., 2022) |
|  | methyl linoleate | A | (Liu et al., 2022) |
|  | methyl (9E)-9-octadecenoate | A | (Liu et al., 2022) |
|  | methyl ricinoleate | A | (Liu et al., 2022) |
|  | methyl stearate | A | (Liu et al., 2022) |
|  | 9(E),11(E)-Conjugated linoleic acid methyl ester | A | (Liu et al., 2022) |

Table 1 (*continued*)

| NO. | Mabolites | Source | References |
| --- | --- | --- | --- |
|  | E-3,7,11,15-Tetramethyl-9-(triisopropyl-silanoxy)-hexadeca-2,6,10,14-tetraen-1-ol | A | (Liu et al., 2022) |
|  | cholesterolcis-11-eicosenoate | A | (Liu et al., 2022) |
|  | 18-Methylnonadecanoic acid methyl ester | A | (Liu et al., 2022) |
|  | heneicosanoic Acid methyl ester | A | (Liu et al., 2022) |
|  | 22-Hydroxy docosanoic acid methyl ester | A | (Liu et al., 2022) |
|  | methyl tricosanoate | A | (Liu et al., 2022) |
|  | methyl Lignocerate | A | (Liu et al., 2022) |
|  | cycloartenol | A | (Liu et al., 2022) |
|  | (6E,10E,14E,18E)-2,6,10,15,19,23-Hexamethyltetracosa-1,6,10,14,18,22-hexaen-3-ol | A | (Liu et al., 2022) |
|  | β-Tocopherol | A | (Liu et al., 2022) |
|  | tetratriacontane | A | (Liu et al., 2022) |
|  | 1-Heptacosanol | A | (Liu et al., 2022) |
|  | vitamin E | A | (Liu et al., 2022) |
|  | stigmasterol | A | (Liu et al., 2022) |
|  | phytyl undecanoate | A | (Liu et al., 2022) |
| **additional identified metabolites** | | | |
|  | isopalmitic acid | A | (Liu et al., 2022) |
|  | oleic acid | A | (Liu et al., 2022) |
|  | myristic acid | A | (Liu et al., 2022) |
|  | 9-Hydroxy-10,12,15-octadecatrienoic acid | A | (Liu et al., 2022) |
|  | stearic acid | A | (Liu et al., 2022) |
|  | arachidic acid | A | (Liu et al., 2022) |
|  | heneicosanoic acid | A | (Liu et al., 2022) |
|  | tricosanoic acid | A | (Liu et al., 2022) |
|  | 12-Oxo-phytodienoic acid | A | (Liu et al., 2022) |
|  | glyceryl linolenate | A | (Liu et al., 2022) |
|  | 2,3-Dihydroxypropyl hexadecanoate | A | (Liu et al., 2022) |
|  | monoolein | A | (Liu et al., 2022) |
|  | monolinolein | A | (Liu et al., 2022) |
|  | α-Linolenic acid | A | (Liu et al., 2022) |
|  | methylpent-2-enoic acid | A | (Liu et al., 2022) |

Table 1 (*continued*)

| NO. | Mabolites | Source | References |
| --- | --- | --- | --- |
|  | 9,10,13-Trihydroxy-11-octadecenoic acid | A | (Liu et al., 2022) |
|  | (+/-)-Jasmonic acid | A | (Liu et al., 2022) |
|  | (+)-Abscisic acid | A | (Liu et al., 2022) |
|  | methyl jasmonate | A | (Liu et al., 2022) |
|  | artemisinic acid | A | (Liu et al., 2022) |
|  | linolenic acid ethyl ester | A | (Liu et al., 2022) |
|  | 5-[(Z)-5-Hydroxy-3-methylpent-3-enyl]-1,4a-dimethyl-6-methylidene-3,4,5,7,8,8a-hexahydro-2H-naphthalene-1-carboxylic acid | A | (Liu et al., 2022) |
|  | [(2S)-2-Benzamido-3-phenylpropyl](2S)-2-benzamido-3-phenylpropanoate | A | (Liu et al., 2022) |
|  | loliolide | A | (Liu et al., 2022) |
|  | scoparone | A | (Liu et al., 2022) |
|  | senkyunolide A | A | (Liu et al., 2022) |
|  | linderalactone | A | (Liu et al., 2022) |
|  | clareolide | A | (Liu et al., 2022) |
|  | (+)-Costunolide | A | (Liu et al., 2022) |
|  | isomeranzin | A | (Liu et al., 2022) |
|  | lovastatin | A | (Liu et al., 2022) |
|  | dibutylphthalate | A | (Liu et al., 2022) |
|  | phenethyl acetate | A | (Liu et al., 2022) |
|  | methyl β-orcinolcarboxylate | A | (Liu et al., 2022) |
|  | 2-Methoxycinnamic acid | A | (Liu et al., 2022) |
|  | erythronolactone | A | (Liu et al., 2022) |
|  | ethyl caffeate | A | (Liu et al., 2022) |
|  | isobutyl 4-hydroxybenzoate | A | (Liu et al., 2022) |
|  | methyl hexadecanoate | A | (Liu et al., 2022) |
|  | di-n-butyl phthalate | A | (Liu et al., 2022) |
|  | ligustilide | A | (Liu et al., 2022) |
|  | glabrolide | A | (Liu et al., 2022) |
|  | 2-[3-(hexadecyloxy)-2-hydroxypropylphosphonic acid]oxyethyl)trimethylammonium LPC 16:0 | A | (Liu et al., 2022) |
|  | phellopterin | A | (Liu et al., 2022) |
|  | 8-(2-Hydroxy-3-methylbut-3-enyl)-7-methoxychro-men-2-one | A | (Liu et al., 2022) |
|  | isoimperatorin | A | (Liu et al., 2022) |
|  | 8-(2-Hydroxy-1-methoxy-3-methylbut-3-enyl)-7-methoxychromen-2-one | A | (Liu et al., 2022) |

Table 1 (*continued*)

| NO. | | Mabolites | Source | References |
| --- | --- | --- | --- | --- |
|  | | scopoletin | A | (Liu et al., 2022) |
|  | | isofraxidin | A | (Liu et al., 2022) |
|  | | 7-Methoxycoumarin | A | (Liu et al., 2022) |
|  | | cumarin | A | (Liu et al., 2022) |
|  | | 5-Methoxypsoralen | A | (Liu et al., 2022) |
|  | | rutamarin | A | (Liu et al., 2022) |
|  | | auraptene | A | (Liu et al., 2022) |
|  | | 4-Methyl-6,7-dihydroxycoumarin | A | (Liu et al., 2022) |
|  | osthole | | A | (Liu et al., 2022) |
|  | cortodoxone | | A | (Liu et al., 2022) |
|  | sclareol | | A | (Liu et al., 2022) |
|  | carveol | | A | (Liu et al., 2022) |
|  | cuminyl alcohol | | A | (Liu et al., 2022) |
|  | 3-(4-Hydroxyphenyl)-1-propanol | | A | (Liu et al., 2022) |
|  | cortisone | | A | (Liu et al., 2022) |
|  | phenanthrene-1-carboxylic acid | | A | (Liu et al., 2022) |
|  | 16-Oxo-19-beyeranoic acid | | A | (Liu et al., 2022) |
|  | phenylpyruvic acid | | A | (Liu et al., 2022) |
|  | benzoic acid | | A | (Liu et al., 2022) |
|  | phenylacetic acid | | A | (Liu et al., 2022) |
|  | 2-Furoic acid | | A | (Liu et al., 2022) |
|  | fumaric acid | | A | (Liu et al., 2022) |
|  | 7-Hydroxy-1,4a-dimethyl-9-oxo-7-propan-2-yl-2,3,4,4b,5,6,10,10a-octahydrophenanthrene-1-carboxylic acid | | A | (Liu et al., 2022) |
|  | (5S,9R)-14-(Hydroxymethyl)-5,9-Dimethyltetracyclo[11.2.1.01,10,04,9]hexadecane-5-carboxylic acid | | A | (Liu et al., 2022) |
|  | gamabufotalin | | A | (Liu et al., 2022) |
|  | (8S,9S,10R,11S,13S,14S,17R)-11,17-Dihydroxy-17-(2-hydroxyacetyl)-10,13-dimethyl-2,6,7,8,9,11,12,14,15,16-decahydro-1H-cyclopenta[a]phenanthren-3-one | | A | (Liu et al., 2022) |
|  | 24-oxahexacyclo[15.5.2.01,18,04,17,05,14,08,13]tetracos-15-en-23-one | | A | (Liu et al., 2022) |
|  | (1R,2R,5S,8R,14R,15R,16S)-16-Hydroxy-1,2,14,17,17-pentamethyl-8-(prop-1-en-2-yl)pentacyclo[11.7.0.02,10,05,9,014,18]icosane-5,15-dicarboxylic acid | | A | (Liu et al., 2022) |
|  | Progesterone | | A | (Liu et al., 2022) |
|  | bufalin | | A | (Liu et al., 2022) |

Table 1 (*continued*)

| NO. | Mabolites | Source | References |
| --- | --- | --- | --- |
|  | estriol | A | (Liu et al., 2022) |
|  | (2S,3R,5R,10R,13R,14S,17S)-2,3,14-Trihydroxy-10,13-dimethyl-17-[(2R,3R)-2,3,6-trihydroxy-6-methylheptan-2-yl]-2,3,4,5,9,11,12,15,16,17-decahydro-1H-cyclopenta[a]phenanthren-6-one | A | (Liu et al., 2022) |
|  | deoxycholic acid | A | (Liu et al., 2022) |
|  | cholic acid | A | (Liu et al., 2022) |
|  | glycocholic acid | A | (Liu et al., 2022) |
|  | sinapoyl aldehyde | A | (Liu et al., 2022) |
|  | atranol | A | (Liu et al., 2022) |
|  | p-Hydroxybenzaldehyde | A | (Liu et al., 2022) |
|  | 3-Hydroxybenzaldehyde | A | (Liu et al., 2022) |
|  | vanillin | A | (Liu et al., 2022) |
|  | 3,5-Dimethoxy-4-hydroxybenzaldehyde | A | (Liu et al., 2022) |
|  | coniferyl aldehyde | A | (Liu et al., 2022) |
|  | cinnamaldehyde | A | (Liu et al., 2022) |
|  | vitamin D2 | A | (Liu et al., 2022) |
|  | pyridoxine | A | (Liu et al., 2022) |
|  | nicotinamide | A | (Liu et al., 2022) |
|  | biotin | A | (Liu et al., 2022) |
|  | nicotinic acid | A | (Liu et al., 2022) |
|  | embelin | A | (Liu et al., 2022) |
|  | keucodin | A | (Liu et al., 2022) |
|  | glycine | A | (Liu et al., 2022) |
|  | kainic acid | A | (Liu et al., 2022) |
|  | L-Phenylalanine | A | (Liu et al., 2022) |
|  | sucrose | A | (Liu et al., 2022) |
|  | sorbose | A | (Liu et al., 2022) |
|  | (2R,3S,4S,5R,6R)-5-[(2S,3R,4R)-3,4-Dihydroxy-4-(hydroxymethyl)oxolan-2-yl]oxy-2-(hydroxymethyl)-6-(2-phenylethoxy)oxane-3,4-diol | A | (Liu et al., 2022) |
|  | benzyl alcohol glycoside | A | (Wu, 2010) |
|  | phenylethanoid glycoside | A | (Wu, 2010) |
|  | (2’S)-2’-3’-dihydroxypropyl-1,6,8-trihydroxy-3-methylanthraquinone-2-carboxylate | A | (Wu, 2010) |

Table 1 (*continued*)

| NO. | Mabolites | Source | References |
| --- | --- | --- | --- |
|  | (3-hydroxy-1-propenyl)-2,6-dimethoxyphenyl-β-D-glucopyranoside | B | (Huang et al., 2012) |
|  | 4-(3-hydroxy-1-propenyl)-2,6-dimethoxyphenyl-β-D-5-glucopyranoside | A | (Liu et al., 2022) |
|  | 3,5-dimethoxybenzyl alcohol 4-O-β-D-glucopyranoside | B | (Huang et al., 2012) |
|  | dihydrophaseic acid 4’-O-β-D-glucopyranoside | B | (Huang et al., 2012) |
|  | uridine | B | (Huang et al., 2012) |
|  | adenosine | B | (Huang et al., 2012) |
|  | succinic anhydride | A | (Li, 2007) |
|  | α-furancarboxylic acid | A | (Li, 2007) |
|  | turformosinic acid | A | (Liu et al., 2022) |
|  | casuaricitin | A | (Li et al., 2015) |
|  | quinic acid | A | (Li et al., 2013) |
|  | β-sitosterol | A;B;C;D | (Li, 2007; Sun, 2008) |
|  | daucosterol | A;B;C;D | (Li, 2007; Sun, 2008) |
|  | 5,10,15-Trimethyl-4,9,13-trioxatetracyclo[10.3.0.03,5.08,10]pentadec-1(15)-en-14-one | A | (Liu et al., 2022) |
|  | cyclo(leucylprolyl) | A | (Liu et al., 2022) |
|  | (-)-12-Hydroxyjasmonic acid | A | (Liu et al., 2022) |
|  | 6-Hydroxyindole | A | (Liu et al., 2022) |
|  | 3-Formylindole | A | (Liu et al., 2022) |
|  | β-D-Glucopyranoside,(3Z)-3-hexen-1-yl | A | (Liu et al., 2022) |
|  | (3E)-4-(1,2,4-Trihydroxy-2,6,6-trimethylcyclohexyl)-3-buten-2-one | A | (Liu et al., 2022) |
|  | 3,8a-Dihydroxy-5-isopropylidene-3,8-dimethyl-2,3,3a, 4,5,8a-hexahydro-6(1H)-azulenone | A | (Liu et al., 2022) |
|  | (1R,2E,7R,10E,12S,13S,15R)-12,15-Dihydroxy-7-methyl-8-oxabicyclo[11.3.0]hexadeca-2,10-dien-9-one | A | (Liu et al., 2022) |
|  | azuleno(5,6-c)furan-1(3H)-one,4,4a,5,6,7,7a,8,9-octahydro-3,4,8-trihydroxy-6,6,8-trimethyl- | A | (Liu et al., 2022) |
|  | (3aR,5S,5aS,9aR)-5,8-Dimethyl-1-methylidene-4,5,5a,6,9,9a-hexahydro-3aH-azuleno[6,5-b]furan-2,7-dione | A | (Liu et al., 2022) |
|  | N-(1-Hydroxy-3-phenylpropan-2-yl)benzamide | A | (Liu et al., 2022) |
|  | rhodiocyanoside A | A | (Liu et al., 2022) |
|  | 4-[2-[(1R,4aS,5R,6R,8aS)-6-Hydroxy-5-(hydroxymethyl)-5,8a-dimethyl-2-methylidene-3,4,4a, 6,7,8-hexahydro-1H-naphthalen-1-yl]-1-hydroxyethyl]-2H-furan-5-one | A | (Liu et al., 2022) |
|  | deoxynivalenol | A | (Liu et al., 2022) |
|  | strophanthidin | A | (Liu et al., 2022) |
|  | pyocyanin | A | (Liu et al., 2022) |

Table 1 (*continued*)

| NO. | Mabolites | Source | References |
| --- | --- | --- | --- |
|  | 4-Hydroxy-4-(3-hydroxybutyl)-3,5,5-trimethylcyclohex-2-en-1-one | A | (Liu et al., 2022) |
|  | gardenin B | A | (Liu et al., 2022) |
|  | digoxigenin | A | (Liu et al., 2022) |
|  | telocinobufagin | A | (Liu et al., 2022) |
|  | (1R,7R)-7-Ethenyl-1,4a,7-trimethyl-3,4,4b,5,6,9,10,10a-octahydro-2H- | A | (Liu et al., 2022) |
|  | phthalic anhydride | A | (Liu et al., 2022) |
|  | 20-Hydroxyecdysone 20,22-acetonide | A | (Liu et al., 2022) |
|  | 1-Phenanthrenecarboxylic acid,7-ethenyl-1,2,3,4,4a,4b,5,6,7,9,10,10a-Dodecahydro-9-hydroxy-1,4a,7-trimethyl- | A | (Liu et al., 2022) |
|  | (1S,4S,5R,10S,13S,17S,19S,20R)-10-Hydroxy-4,5,9,9,13,19,20-heptamethyl- | A | (Liu et al., 2022) |
|  | 1,7-Diphenylhept-4-en-24-one | A | (Liu et al., 2022) |
|  | 1,7-Dimethyl-7-(4-methyl-3-penten-1-yl)bicyclo[2.2.1]heptan-2-ol | A | (Liu et al., 2022) |
|  | paracetamol | A | (Liu et al., 2022) |
|  | 4-Aminobenzoate | A | (Liu et al., 2022) |
|  | theviridoside | A | (Liu et al., 2022) |
|  | ginsenoside-Rg1 | A | (Liu et al., 2022) |
|  | tyramine | A | (Liu et al., 2022) |
|  | matairesinol | A | (Liu et al., 2022) |
|  | syringaresinol | A | (Liu et al., 2022) |
|  | sodium lauryl sulfate C12-AS | A | (Liu et al., 2022) |
|  | 1,7-bis(4-Hydroxyphenyl)heptan-3-one | A | (Liu et al., 2022) |
|  | 13-Hotre | A | (Liu et al., 2022) |
|  | (E)-5-(2,3-Dimethyl-4,5,6,7-tetrahydro-1H-tricyclo[2.2.1.02,6]heptan-3-yl)-2- | A | (Liu et al., 2022) |
|  | idroxioleic acid | A | (Liu et al., 2022) |
|  | p-hydroxycinnamic acid | A;B;C;D | (Sun et al., 2012) |

Note: A: Leaves; B: Stem; C: Bark; D: Branch.
